# The FleQ-FleN circuit balances flagellar number against fitness in *Pseudomonas aeruginosa*

**DOI:** 10.1128/mbio.00360-26

**Published:** 2026-08-05

**Authors:** Anali Migueles-Lozano, Merrill Asp, Sofia T. Rocha, Jiaqi Li, Georgia Fanouraki, Aden D. Sun, Lichun Zhang, Jacob R. Waldbauer, Jiarong Hong, Abhishek Shrivastava, Jing Yan, Sampriti Mukherjee

**Affiliations:** 1 Department of Molecular Genetics & Cell Biology, University of Chicago 311543, Chicago, Illinois, USA; 2 Department of Molecular, Cellular and Developmental Biology, Yale University 118551, New Haven, Connecticut, USA; 3 Yale Quantitative Biology Institute, Yale University 5755, New Haven, Connecticut, USA; 4 School of Life Sciences, Arizona State University 69991, Tempe, Arizona, USA; 5 Biodesign Institute, Arizona State University 43363, Tempe, Arizona, USA; 6 Department of Mechanical Engineering, University of Minnesota 172311, Minneapolis, Minnesota, USA; 7 Department of the Geophysical Sciences, University of Chicago 166952, Chicago, Illinois, USA; 8 Department of Microbiology, University of Chicago 456539, Chicago, Illinois, USA; 9 Duchossois Family Institute, University of Chicago 2462, Chicago, Illinois, USA; Gulbenkian Institute for Molecular Medicine, Oeiras, Portugal

**Keywords:** flagellar gene regulation, *Pseudomonas*, biofilms, adaptive fitness, virulence, evolution

## Abstract

Evolutionary constraints governing flagellar number in bacterial pathogens remain poorly understood. While related *Pseudomonas* species are hyperflagellated, *Pseudomonas aeruginosa* maintains strict monoflagellation through the FleQ-FleN regulatory circuit. Here, we demonstrate that FleN dosage is essential for maintaining monoflagellation and fitness. Confirming earlier reports, wild-type *P. aeruginosa* displayed unipolar monoflagellation, whereas ∆*fleN* mutants developed multiple flagella, frequently more than four, in unipolar or bipolar arrangements. These hyperflagellated cells showed marked fitness defects, including reduced growth, attenuated virulence in a nematode infection model, and a competitive disadvantage in co-culture. The virulence defect reflected hyperflagellation rather than the loss of motility, since a non-flagellated ∆*fliC* mutant retained full pathogenicity. Notably, ∆*fleN* cells rapidly evolved suppressor mutations in *fleQ* that partially restored growth and motility without always restoring monoflagellation. Five independent suppressor alleles mapped to key FleQ domains, four in the AAA+ ATPase domain, and one in the DNA-binding domain, consistent with reduced FleQ activity that rebalances the circuit. Single-cell holographic tracking showed that suppressor strains swim with heterogeneous dynamics, including subpopulations that reach wild-type (WT) speeds, despite carrying multiple flagella. Quantitative proteomics indicated that the fitness burden extends beyond flagellar components, with protein-abundance changes across metabolism, stress responses, and signaling. Conversely, selection under high-viscosity conditions drove wild-type cells to acquire enhanced spreading through a recurrent *fleN* mutation, *fleN*^*V178G*^; multiflagellation is therefore accessible. Together, these findings indicate that the FleQ-FleN circuit balances the flagellar number against fitness, holding *P. aeruginosa* at a single flagellum while retaining the latent capacity to reach multiflagellated states through single mutations.

**IMPORTANCE:**

Bacterial flagella are extracellular appendages that rotate to propel the cell and enable swimming motility. While some bacteria have multiple flagella, many pathogenic species like *Pseudomonas aeruginosa* have just one. Surprisingly, mutants of *P. aeruginosa* with multiple flagella performed worse, that is, they grew more slowly, were less infectious in laboratory animals, and were outcompeted by wild-type bacteria. Even when some mutant bacteria evolved compensatory changes, they still struggled compared to single-flagellum bacteria. This reveals an important evolutionary trade-off: while multiple flagella might seem advantageous for movement, having just one flagellum allows the bacteria to grow faster and cause more severe infections. This plasticity likely explains why *P. aeruginosa* is so successful both in the environment and as a human pathogen.

## INTRODUCTION

The specification of appendage number and location is a fundamental biological requirement across all domains of life. Through evolutionary processes, organisms develop specific arrangements and functions (such as feeding and locomotion) of these appendages to optimize survival in their particular environments (1, 2). Evolutionary changes in appendage number are common, even within the same phylogenetic order—for instance, only 40% of Phasmatodea insects (stick insects) possess full wings, with the remainder being partially or completely wingless (3–5). This precise maintenance of appendage patterns across generations proves equally vital for microorganisms such as bacteria, particularly for their various motility mechanisms: swimming, swarming, gliding, or twitching. The locomotion and maneuverability of microorganisms are central to their fitness, with species-specific motility differences determining population and community-level outcomes as movement capabilities fundamentally shape how cells navigate their environments in search of resources and favorable conditions (6–9). Flagellar motility, in particular, enables rapid swimming and precise chemotactic responses, allowing bacteria to detect and move toward nutrients or away from toxins along chemical gradients (10–13). This ability to respond to environmental cues through directed movement influences microbial distribution patterns, biofilm formation, and interspecies competition. Beyond nutrient acquisition, flagellar motility plays critical roles in host colonization, pathogenesis, and dispersal, directly impacting transmission dynamics and infection processes (14–17). However, there are significant energetic costs of construction and operation of flagella due to the elaborate building of this multimodule nanomachine (18–21).

A bacterial flagellum is assembled from over 30 proteins in three architectural domains: the basal body anchoring the flagellum to the cell envelope, the hook universal joint, and the helical propeller-like filament (22–25). Bacteria exhibit diverse flagellar arrangements including monotrichous (single polar), lophotrichous (multiple at one pole), peritrichous (distributed around cell), and amphitrichous (at both poles), which produce distinct swimming patterns (run-tumble, run-reverse, and wrapping), diversifying exploration strategies and resource access (10, 26–28). The peritrichous arrangement is exemplified by *Escherichia coli*, which typically possesses 6–10 flagella randomly distributed across its cell surface, enabling the characteristic run-and-tumble motility pattern essential for chemotaxis and environmental exploration. In the polar flagellates, diversity in flagellar number is exemplified within *Pseudomonas*: *Pseudomonas aeruginosa* maintains a single unsheathed polar flagellum, *Pseudomonas putida* typically possesses 5–7 polar flagella per cell, *Pseudomonas fluorescens* exhibits strain-specific variations from two flagella (MFE01) to a single flagellum (SBW25) to seven flagella (C7R12), whereas *Pseudomonas syringae* averages 2.7 flagella per cell with notable stochastic variation and bimodal expression patterns (29). However, key questions remain: why do bacterial species exhibit such flagellar variation, what evolutionary and ecological factors drive monotrichous versus lophotrichous systems, and how do bacteria balance flagellar biosynthesis and operation costs against mobility advantages in different environments?

Here, we addressed these questions using the monotrichous versatile bacterium *P. aeruginosa,* which can be either pathogenic in humans (30, 31) or beneficial/harmful in plants (32, 33), with flagellar motility being crucial for its colonization capabilities. Flagellar motility in *P. aeruginosa* directly correlates with increased pneumonia and burn wound sepsis mortality rates (34–37). Unlike related *Pseudomonas* species with multiple flagella, monotrichous *P. aeruginosa* is thought to navigate using a “run-reverse” strategy (26). Flagellar assembly is regulated by the master transcription regulator, FleQ, which was first identified and characterized by Arora and colleagues in the late 1990s (38). FleQ has been classified as an atypical enhancer-binding protein (EBP) from the NtrC family of transcription factors (38–41). Different from traditional NtrC EBPs, FleQ lacks the aspartic acid residue that histidine kinases phosphorylate to activate conventional EBPs (38), and FleQ can both repress and activate transcription by binding DNA upstream or downstream of the targeted genes (39, 42, 43). Transcriptional regulation of fleQ has been identified to be dependent on σ^70^ and negatively regulated by Vfr (44) and the AlgU-dependent transcriptional regulator AmrZ (45). FleQ protein has three distinct domains: regulatory, AAA+ ATPase, and DNA-binding domains (46, 47). The AAA+ ATPase domain is a major binding site for FleQ activity regulators. Unlike typical NtrC transcription factors, the tuning of FleQ activity does not require phosphorylation; instead, it is mediated by the binding of cyclic di-GMP (c-di-GMP) (43, 47–50) and its antiactivator, FleN (46, 51–54), to FleQ’s AAA+ domain. FleQ-FleN interaction has been widely studied, and the current models suggest that FleN regulation goes beyond inhibiting FleQ ATPase activity; rather, FleN stabilizes FleQ conformational and stoichiometry states, promoting the dual role of FleQ as an activator and repressor, together with c-di-GMP levels (46, 48, 52).

**Fig 1:**
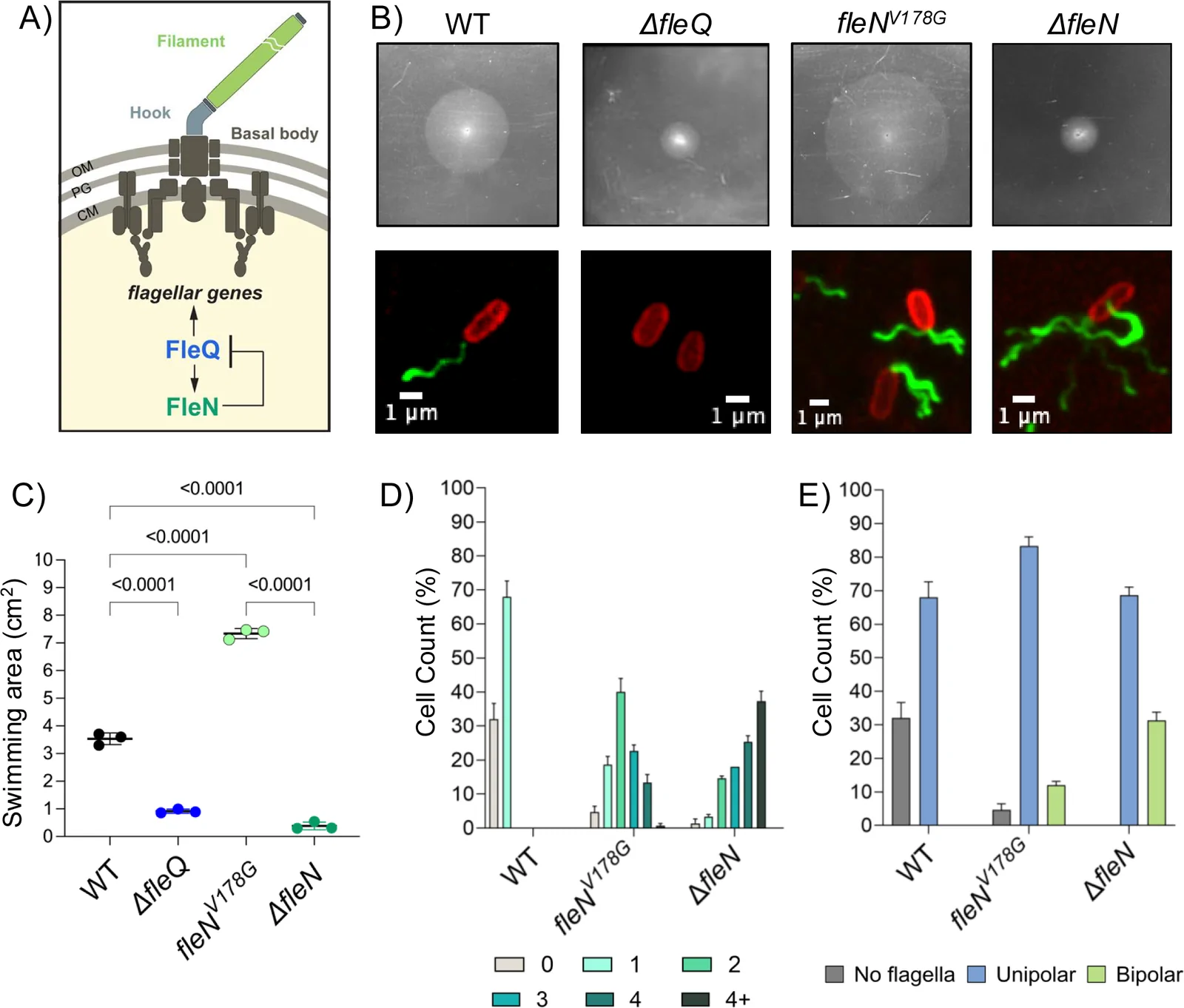
Flagellar number and patterning in WT and *fleN* mutant strains. (**A**) Schematic of the conserved flagellar architectural domains and the FleQ-FleN regulatory axis. FleQ is the master transcriptional factor of flagellar genes, and its activity is directly inhibited by the anti-activator FleN. FleQ promotes the expression of FleN. (**B**) Top row: representative images of swimming phenotype in low agar concentration plates (0.3%) of (from left to right) WT, ∆*fleQ, fleN^V178G^*, and ∆*fleN* strains. Bottom row: representative images of flagellar patterning of (from left to right) WT, ∆*fleQ, fleN^V178G^*, and ∆*fleN*. Flagellar filament and membrane stained using Alexa488 (green) and membrane dye (FM4-64), respectively. (**C**) Swimming area measurement of WT, ∆*fleQ, fleN^V178G^*, and ∆*fleN* strains. Error bars represent the SD of three biological replicates. Statistical significance was determined using one-way ANOVA (R^2^ = 0.998, *P* < 0.0001), followed by a Tukey’s *post-hoc* multiple comparisons test in GraphPad Prism software. All mutant strains differed significantly from the WT control: ∆*fleN* (M_diff_ = 3.15, 95% CI [2.73, 3.57]), *fleN^V178G^* (M_diff_ = −3.80, 95% CI [−4.22, −3.38]), ∆*fleQ* (M_diff_ = 2.62, 95% CI [2.20, 3.04]); ****P < 0.0001 for all. ∆*fleN* and *fleN^V178G^* mutants (M_diff_ = −6.96, 95% CI [−7.38, −6.54], ****P < 0.0001). The remaining inter-mutant comparisons were also statistically significant (*P* < 0.05) but not displayed in the graph. (**D**) Percentage of cells exhibiting varying numbers of flagella during the exponential growth phase (OD_600_ = 0.5) in WT, ∆*fleQ, fleN^V178G^*, and ∆*fleN* strains; 50 cells per replicate, *N* = 3. Gray, non-flagellated; cyan, 1; turquoise, 2; dark turquoise, 3; emerald green, 4; dark green: More than four flagella per cell. (**E**) Percentage of cells with different flagellar placement from the cells in panel **D**.

In *P. aeruginosa*, FleQ regulates the contrasting motile (flagellum-based) and sessile (biofilm) lifestyles, and the transition between them (43, 47, 48). During the motile lifestyle, FleQ positively regulates flagellar assembly through a sophisticated four-tiered hierarchical cascade: FleQ (class I) activates class II genes in a σ^54^-dependent manner; FleSR (55) (class II) activates class III genes for hook-basal body assembly. Once this structure is complete, FlgM is exported, releasing FliA (σ^28^) to activate class IV genes encoding the flagellar filament and motor proteins (41, 56). FleQ activity is precisely controlled by the anti-activator FleN (Fig. 1A) (46, 51, 52, 54, 57), whose expression is promoted by the FleQ cascade and which binds to FleQ’s ATPase domain, tuning down the transcription of flagellar genes. In contrast to the motile lifestyle, the cells can also form sessile biofilm communities. Biofilms are more tolerant to the host immune response and antimicrobial agents; hence, the prevalence of chronic infections is associated with this sessile lifestyle. Furthermore, biofilms are also characterized by the production of an extracellular polymeric matrix. FleQ is one of the main mediators of the transitions between motile and sessile lifestyles (42, 43, 47, 58). Under conditions of low intracellular c-di-GMP, FleQ activates σ⁵⁴-dependent transcription of flagellar genes while simultaneously repressing exopolysaccharide biosynthesis operons (*pel* and *psl*). In contrast, elevated c-di-GMP levels relieve this repression, promoting biofilm matrix production (50).

In this study, we investigated the fitness consequences of hyperflagellation, that is, whether there is an evolutionary trade-off between flagellar number and growth/virulence. We demonstrate that although *P. aeruginosa* can develop multiple flagella, this hyperflagellation comes with significant fitness costs, including reduced growth, competitiveness, and virulence. These findings suggest that the regulatory network governing flagellar assembly may function as an evolutionary capacitor—a system that normally constrains flagellar number to optimize fitness while maintaining the latent capacity for phenotypic diversification. Under standard conditions, this regulatory architecture buffers against costly hyperflagellation, but the retention of this developmental plasticity permits rapid phenotypic exploration when environmental pressures favor alternative flagellation states. Thus, *P. aeruginosa* appears to have evolved regulatory plasticity in its flagellar assembly cascade as an adaptive strategy that balances phenotypic robustness against rapid adaptation across its fluctuating ecological niches and pathogenic lifestyle.

## RESULTS

### Wild-type *P. aeruginosa* PA14 exhibits robust monotrichous flagellation

The fact that the wild-type (WT) *P. aeruginosa* UCBPP-PA14 (hereafter called PA14) strain is monoflagellated was established earlier by electron microscopy (14); here, we resolved, at single-cell resolution and across growth, the full per-cell distribution of flagellar number together with its polarity, which the earlier electron-microscopy snapshots had not provided. To visualize and count the number of completed flagella, we introduced into the *fliC* gene, encoding for the filament protein flagellin, a cysteine mutation (*fliC^T394C^*) that allowed labeling with thiol-reactive fluorescent Alexa Fluor 488 dye without affecting swimming motility (59, 60) (Fig. 1B). We monitored filament number using confocal microscopy at different growth stages (Fig. 1B, D, and E; Fig. S1). During the early exponential phase (OD_600_ = 0.25), the cells displayed mixed flagellation patterns: 51.33% monotrichous, 48% non-flagellated, and rarely (0.66%) multiple flagella clustered at one pole, while from the exponential through the stationary phase (OD_600_ = 0.5–1.5), monotrichous flagellation predominated (68%–82%) (Fig. S1). We conclude that WT PA14 maintains a predominantly unipolar, single-flagellum pattern over the course of growth under planktonic conditions, refining the earlier qualitative counts into a growth-resolved distribution of flagellar number and placement.

*P. aeruginosa* encounters dramatically different physical environments throughout its complex lifecycle, from the watery surfaces in natural habitats to the viscous mucus of cystic fibrosis airways (31, 32, 34, 61–63). This environmental heterogeneity creates fluctuating demands on bacterial motility systems, where optimal flagellar configurations must balance swimming efficiency against metabolic costs under varying physical constraints (18, 61, 64–68). To investigate whether environmental pressures could drive the evolution of alternative flagellar configurations, we subjected WT *P. aeruginosa* PA14 to selection on viscous swimming medium (0.35% agar compared to standard 0.3%) that increases motility demands. After extended incubation (48 h at 37°C), rare but consistent outgrowth flares emerged from the primary inoculation zones, indicating cells with improved swimming ability under viscous conditions. Sequencing analysis of eight independent laboratory-evolved isolates revealed a striking pattern: all mutants harbored the identical mutation, *fleN^V178G^*, that has been previously identified through swarming selection (14). Characterization of PA14 *fleN*^V178G^ cells confirmed enhanced swimming motility in soft agar plates compared to WT, while the assessment of flagellar number and polarity showed increased flagellar numbers per cell and a subpopulation of cells exhibiting amphitrichous hyperflagellation (Fig. 1B through E). Furthermore, in comparison with the Δ*fleN* strain, which is deficient in biofilm formation, the PA14 *fleN*^V178G^ strain can form biofilms, yet does not reach wild-type levels (*P*-value: 0.0185) (Fig. S2D). This remarkable convergence on a single amino acid, that is, position 178 in FleN, represents a critical regulatory hotspot, enabling evolutionary fine-tuning of the flagellar output.

Next, we investigated the broader evolutionary potential of the FleN-FleQ regulatory circuit. To explore the full range of possible flagellar phenotypes due to FleN activity, we constructed an in-frame markerless deletion of *fleN* and quantified the resulting phenotypes. In the absence of FleN, FleQ activity is unchecked, giving rise to cells with a hyperflagellation phenotype (Fig. 1B, D and E; Fig. S1). At the early growth stage (OD_600_ = 0.25), there are cells having zero (1.33%), one (10.6%), two (16.66%), three (22.66%), four (21.33%), and more than four (27.33%) flagella (Fig. S1). From exponential growth through the stationary phase (OD_600_ = 0.5–1.5), the fraction of cells having more than four flagella became the predominant phenotype (37.33%–50.66%). Furthermore, Δ*fleN* cells displayed three different types of flagella arrangements: monotrichous (single polar flagellum), amphitrichous (flagella at both poles), and lophotrichous (cluster of flagella at one end), each at different frequencies, lophotrichous being the predominant one (>50% in every growth stage) (Fig. 1B through E; Fig. S1). Through exponential growth, the fraction of cells having flagella at both poles increased; from 16.66% to 31.33% during the exponential phase (OD_600_ = 0.25 and 0.5) to 42.66% at the stationary phase (OD_600_ = 1.5).

**Fig 2:**
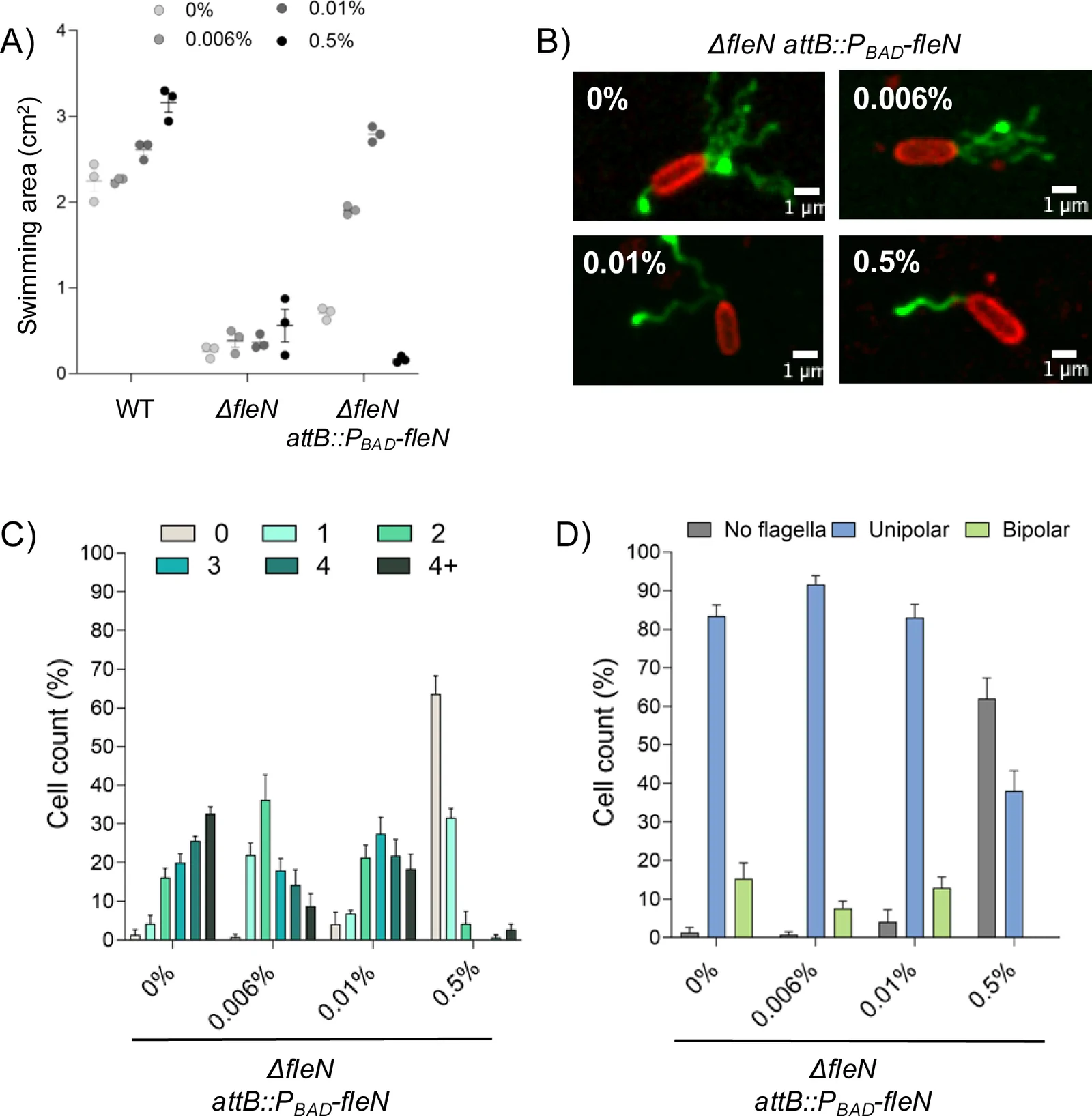
FleN dosage controls flagellar number and swimming motility. (**A**) Swimming area measurement of WT, ∆*fleN,* and ∆*fleN attB::P_BAD_-fleN* strains, using 0%, 0.006%, 0.01%, and 0.5% concentrations of arabinose. Error bars represent the SD of three biological replicates. (**B**) Representative images of flagellar patterning of the ∆*fleN attB::P_BAD_-fleN* strain at the indicated arabinose concentrations. Flagellar filament and membrane stained using Alexa488 (green) and membrane dye (FM4-64), respectively. (**C**) Percentage of cells exhibiting varying numbers of flagella during the exponential growth phase (OD_600_ = 0.5) in the ∆*fleN attB::P_BAD_-fleN* strain at the indicated arabinose concentrations, 50 cells per replicate, *n* = 3. Gray, non-flagellated; cyan, 1; turquoise, 2; dark turquoise, 3; emerald green, 4; dark green: More than four flagella per cell. (**D**) Percentage of cells with different flagellar placement from the cells in panel **C**. Error bars for **C** and **D** represent the SEM of 50 cells per replica and three biological replicates. The 0% arabinose concentration values shown in the plot were calculated by averaging the 0% negative controls for each condition, since each concentration had a separate 0% negative control.

Despite producing multiple flagella, ∆*fleN* cells showed severely compromised swimming motility in soft agar assays, with swimming zone diameters decreased by approximately 70% compared to WT (Fig. 1B and C). This counterintuitive result demonstrated that flagellar number alone does not determine swimming performance; instead, proper flagellar arrangement and coordination are essential for efficient propulsion. Furthermore, this finding provided crucial context for understanding why the V178G mutation, which increases flagellar number only modestly, provides adaptive advantages, while the absence of FleN impairs function. To confirm that the observed phenotypes resulted specifically from *fleN* loss, we complemented ∆*fleN* cells using an arabinose-inducible *fleN* construct integrated at the chromosomal *attB* site. Arabinose titration experiments (0.006%–0.5%) revealed a complex dose-response relationship with distinct threshold effects (Fig. 2A). Low arabinose concentrations (0.01%) restored swimming motility to WT levels, demonstrating functional complementation (Fig. 2A). However, flagellar number and patterning remained partially altered compared to WT cells, with 18.4% bearing more than four flagella compared to <5% in WT cells (Fig. 2B through D). Flagellar polarity between the arabinose-induced (unipolar 82.97% and bipolar 12.91%) and uninduced (unipolar 84.66% and bipolar 15.33%) conditions had a subtle decrease in bipolar flagella. Notably, high arabinose concentrations (0.5%) completely abolished swimming motility and triggered a dramatic shift toward non-flagellated cells (62% with zero flagella, 30.7% with one flagellum) (Fig. 2B through D). This bifurcation demonstrates that FleN overexpression blocks flagellar assembly entirely, establishing that precise FleN dosage is critical for normal flagellar regulation. The narrow concentration window supporting normal motility indicates that the FleN-FleQ regulatory circuit operates near a critical threshold, making it sensitive to regulatory perturbations.

### Selection pressure drives convergent evolution of *fleQ* suppressor mutations

**Fig 3:**
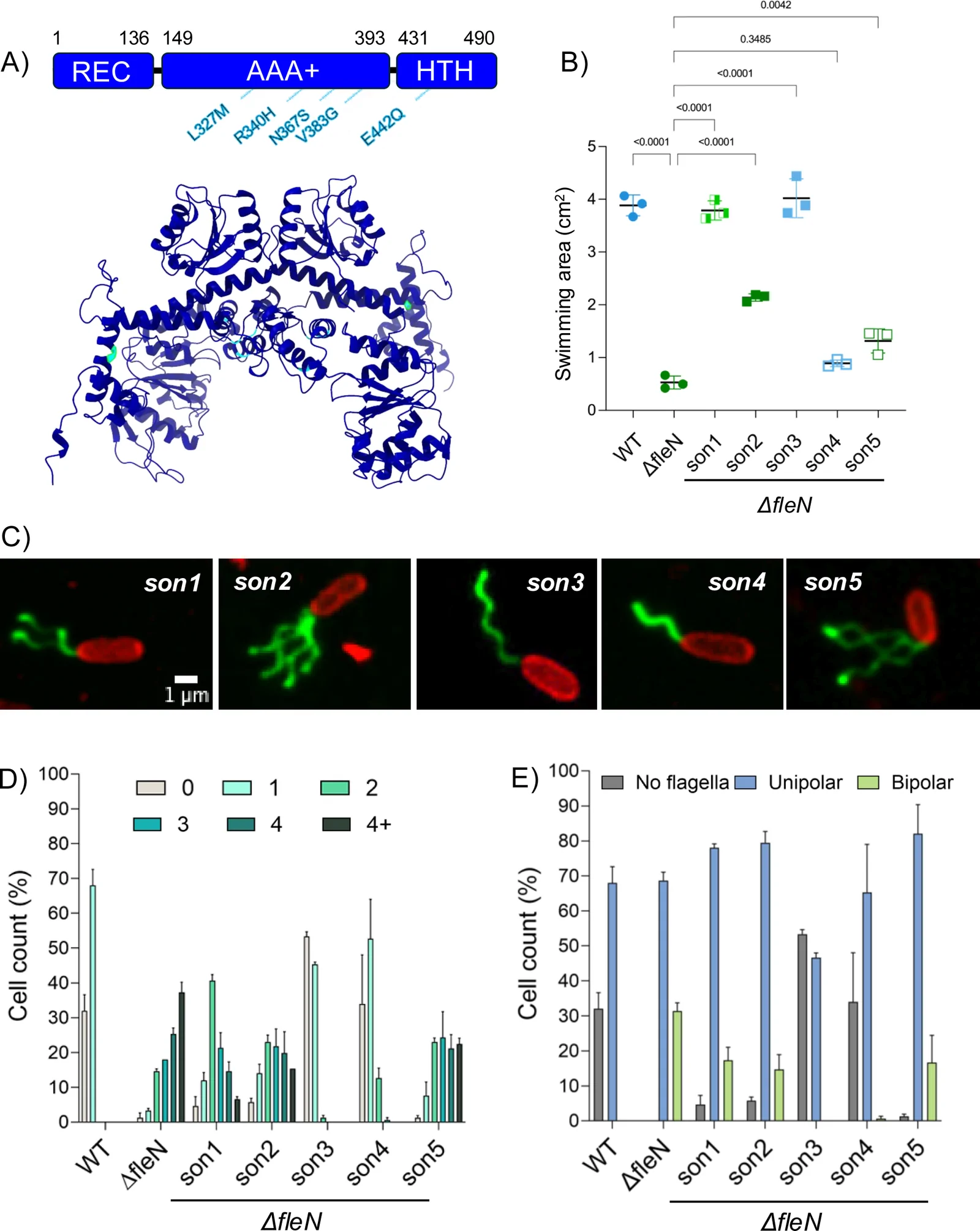
∆*fleN* cells develop suppressor mutations in *fleQ* that restore motility, but not the number of flagella. (**A**) Box (up) and AlphaFold3 (down) representation of the FleQ protein. In the box diagram and AlphaFold3 models, Δ*fleN* suppressor mutations in *fleQ* are denoted in cyan. AlphaFold3 model of FleQ dimer (navy blue). (**B**) Swimming area measurement of the indicated strains. Error bars represent the SD of three biological replicates. Statistical significance was determined using one-way ANOVA (*R^2^* = 0.99, *P* < 0.0001), followed by a Tukey’s *post-hoc* multiple comparisons test in GraphPad Prism software. All pairwise comparisons were statistically significant (*P* < 0.05), except for WT vs *son3* (*P* = 0.9779), WT vs *son1* (*P* = 0.9964), *son1* vs *son3* (*P* = 0.7888), *son4* vs *son5* (*P* = 0.2055), and Δ*fleN* vs *son4* (*P* = 0.3485). (**C**) Representative images of flagellar patterning of the indicated strains: *son1* (*fleQ^L327M^*), *son2* (*fleQ^R340H^*), *son3* (*fleQ^N367S^*), *son4* (*fleQ^V383G^*), and *son5* (*fleQ^E442Q^*). Flagellar filament and membrane stained using Alexa488 (green) and membrane dye (FM4-64), respectively. (**D**) Percentage of cells exhibiting varying numbers of flagella during the exponential growth phase (OD_600_ = 0.5) in the indicated strains, 50 cells per replicate, *n* = 3. Gray, non-flagellated; cyan, 1; turquoise, 2; dark turquoise, 3; emerald green, 4; eark green: More than four flagella per cell. (**E**) Percentage of cells with different flagellar placement from the cells in panel **D**. Error bars for panels **D** and **E** represent the SD of three biological replicas.

To investigate how ∆*fleN* cells could overcome their motility defects, we performed adaptive evolution experiments by plating ∆*fleN* cultures on 0.3% swimming agar and selecting for enhanced motility over 96 h at 37°C. This selection consistently yielded distinct outgrowth flares containing cells with improved swimming ability compared to the parental ∆*fleN* strain, suggesting rapid adaptive evolution. We isolated and characterized 10 independent suppressor strains that demonstrated reproducibly enhanced swimming motility (Table S1). Sequencing analysis revealed a striking pattern: all these suppressors (hereafter referred to as ***s***uppressors ***o***f ∆*fle**N***, i.e., *son*) contained single-nucleotide changes exclusively in *fleQ*, suggesting that the FleN-FleQ regulatory circuit represents an evolutionary hotspot for motility adaptation (Fig. 3A). Four mutations (L327M [*son1*], R340H [*son2*], N367S [*son3*], V383G [*son4*]) clustered within the AAA+ ATPase domain essential for ATP hydrolysis and protein-protein interactions, while one mutation (E442Q, *son5*) localized to the C-terminal helix-turn-helix DNA-binding domain required for target gene recognition (Fig. 3A). Based on this distribution, we hypothesized that suppression of motility defect occurred through impaired FleQ function. To test whether these mutations caused partial loss of FleQ function, we introduced each *fleQ* allele into WT PA14 and assessed swimming motility. All five mutations either decreased motility or showed no significant effect compared to WT, confirming partial loss-of-function properties (Fig. S2A and B). However, when *fleN* was deleted in each *fleQ^son^* mutant background, motility defects were enhanced relative to WT, demonstrating that these mutations provide suppression specifically in the sensitized ∆*fleN* context (Fig. 3B; Fig. S2A and B). Western blot analysis using custom-raised antibodies against FleQ showed that the protein levels of the WT and FleQ^son^ mutants were comparable, thereby ruling out protein stability defects (Fig. S5). We conclude that these suppressor mutations restore regulatory balance by reducing FleQ activity to compensate for the absence of FleN-mediated inhibition.

Quantitative flagellar number and arrangement analysis of the five suppressor strains during exponential growth revealed two phenotypic classes of suppressors—class I mutants exemplified by *son3* and *son4* showed near-complete restoration of WT flagellar numbers with predominantly monotrichous cells, while class II mutants exemplified by *son1*, *son2,* and *son5* retained varying degrees of hyperflagellation (Fig. 3C through E; Fig. S3). Nonetheless, all suppressor strains shared common features distinguishing them from the ancestral ∆*fleN* strain: reduced frequency of bipolar flagellation and decreased proportion of cells with more than four flagella (Fig. 3D and E). This convergent phenotypic pattern indicates that excessive flagellar assembly, particularly at multiple cellular poles, represents the primary constraint limiting motility in hyperflagellated cells. We note that motility in 0.3% agar plates showed a disconnection with flagellar number; for instance, *son4* cells, despite achieving WT flagellar counts, demonstrated minimal improvement in swimming motility compared to the parental ∆*fleN* strain. Conversely, *son1* cells maintained significant hyperflagellation yet achieved swimming speeds comparable to WT. We performed RT-qPCR to determine the consequences of the suppressor mutations on flagellar gene transcription. The levels of *fliF* (class II) transcripts in the *son* mutants were comparable to the original ∆*fleN* strain, except for *son3*, which showed decreased *fliF* expression when compared to WT (Fig. S4A and B). This reduction in the expression of *fliF* in the *son3* mutant population is likely due to more than 50% of the cells lacking flagella (Fig. 3D).

### Single-cell analysis reveals population heterogeneity in swimming dynamics

**Fig 4:**
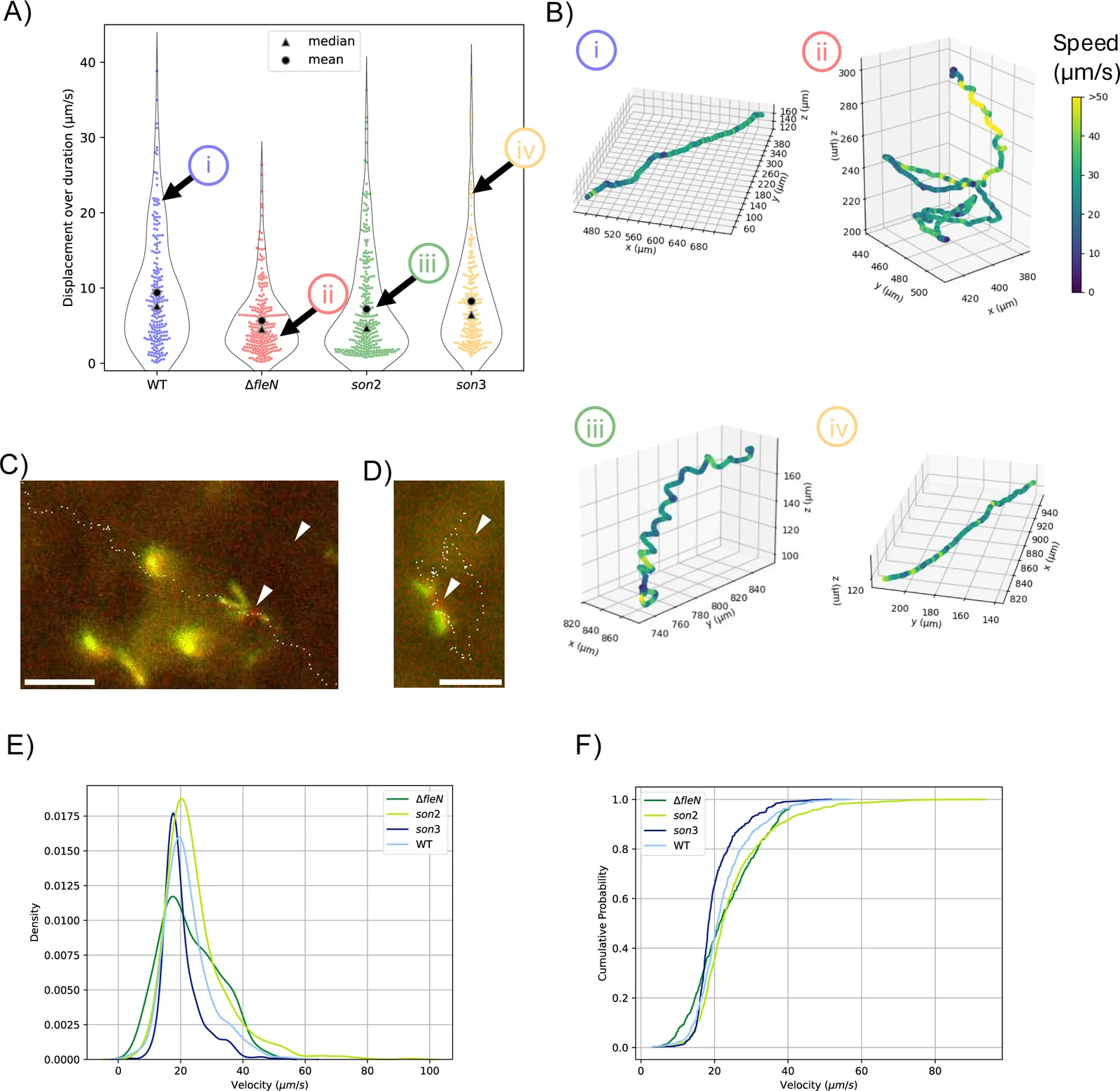
Three-dimensional holographic tracking of single-cell dynamics shows heterogeneous swimming behaviors. (**A**) Displacement over time (the average speed that a cell can locomote from one position to another) is calculated for individual cell trajectories by dividing straight-line end-to-end distance during swimming for up to 15 s per cell. Each point represents an individual cell, with data collected across two biological replicates. (**B**) Three-dimensional trajectories are displayed for four individual cells with mean speeds pointed out as i–iv in panel **A**. Color indicates instantaneous speed. (i) A straight WT trajectory, showing typical high mean speed behavior. (ii) A low mean speed Δ*fleN* trajectory, showing both a helical path (upper) and a meandering path (lower). (iii) A low mean speed *son2* trajectory showing strong helicity. (iv) A high mean speed *son3* trajectory, showing recovery of swimming in a straight line. (**C**) A *son2* cell (cell body in red, constitutively fluorescent) with unbundled flagella (green) on its left side, with both channels imaged simultaneously while the cell is swimming. A small point of green on the right side indicates flagella on the opposite pole, possibly wrapped around the cell. White dots show the progress of the cell body over 3.3 s of swimming, displaying a directed helical trajectory. Scale bar 10 µm. (**D**) Another *son2* cell with flagella active on both poles, undergoing a meandering trajectory, as shown by the white dots that mark the cell body position during 3.8 s of swimming. Scale bar 10 µm. (**E and F**) Distribution of the single cells’ behaviors across the different strains in terms of E average velocity, given by total path length divided by duration for each trajectory, displayed as a probability distribution function smoothed using kernel density estimation (KDE) and F the same average velocity distribution displayed as a cumulative distribution function.

To understand how different flagellar configurations affect individual cell motility, we tracked the centroid positions of individual cells swimming in liquid media across PA14, ∆*fleN*, *son2,* and *son3* strains. We selected *son2* and *son3* as representatives of the two phenotypic classes defined above, with *son2* retaining hyperflagellation and *son3* restoring near-wild-type monotrichous flagellation. Over one thousand quantitative, three-dimensional trajectories were captured with digital in-line holography (69), with several hundred per strain, as detailed in Methods. This revealed typical instantaneous speeds of swimming cells and the shape of their trajectories while tracked over a 15 s time frame. The effective speed (displacement divided by time within the tracked window) reflects both the instantaneous speed and how straight or meandering the cell’s path is. WT PA14 cells exhibited characteristic straight swimming patterns with typical mean speeds of 10 μm/s and the top 5% of mean speeds in excess of 24 μm/s, under the particular conditions we tested. Across all strains, a consistently observable subpopulation remained less motile (Fig. 4A). The ∆*fleN* mutant strain showed dramatically altered swimming dynamics, with highly helical and meandering trajectories that reduced typical mean speeds to about 6 μm/s, with the top 5% of mean speeds only 15 μm/s or above (Fig. 4B). In the *son3* suppressor mutant, trajectory shapes were mostly composed of straight lines, consistent with the monotrichous behavior of WT cells. The effective speeds of these cells closely resembled those of the WT rather than ∆*fleN*, although a subpopulation of less motile cells remained. The per-trajectory average speed across strains showed the same pattern, with the *son2* and *son3* distributions shifting away from ∆*fleN* and toward the WT swimming behavior (Fig. 4E and F). Analysis of trajectory shape across the population revealed significant heterogeneity, with a subset of ∆*fleN* cells achieving WT swimming patterns (relatively straight trajectories at 10 μm/s or more) despite the overall population deficit, consistent with the observation that ∆*fleN* cells show reduced, but not abolished, motility on swimming plates. The helical and meandering trajectories were almost never observed in the WT population, suggesting that miscoordination between multiple flagella may cause less efficient swimming patterns. This finding indicates that hyperflagellation generates phenotypic variation within isogenic populations, generating cells with distinct motility capabilities.

We further supplemented the centroid trajectory data with direct observation of swimming cells’ flagella by employing simultaneous dual-color fluorescence microscopy and capturing videos, with details included in the Methods. This produced several illustrative examples of how hyperflagellation impacts individual cell motility, including helical trajectories that were consistent with hyperflagellated cells (Fig. 4C), and the most meandering trajectories were representatives of bipolar hyperflagellated cells (Fig. 4D). Flagella dynamically bundle and unbundle during swimming, affecting direction and speed, although tight flagellar bundles were observed to not be necessary for swimming as in other multiflagellated species such as *E. coli*. Hyperflagellated cells were occasionally observed swimming faster than typical WT cells, briefly achieving speeds up to 50 μm/s in one instance. Cells with multiple flagella swim best when only flagella on one pole are active, although cells with flagella on the opposite pole were observed wrapping around the cell to achieve persistent motion as well. Overall, increasing the number of flagella drastically increases the variety of swimming modes available to cells, which can switch stochastically over time between different behaviors even for individual cells. The *son2* suppressor mutant demonstrated its own distinct distribution of behaviors, with helical trajectories and typical mean speeds similar to ∆*fleN* but expanded subpopulations of both fast-swimming cells and slow-swimming cells, pushing the top 5% of mean speeds back up to 22 μm/s or more. Notably, analysis of the velocity per trajectory for WT, ∆*fleN*, *and son2* mutants showed the shift toward WT swimming behavior in the *son2* mutant (Fig. 4E and F). Taken together, we conclude that rather than uniform improvement across the population, the *son2* mutant appears to have increased the fraction of cells capable of overcoming hyperflagellation constraints, and the *son3* mutant increased the fraction of cells that swim in a wild-type pattern of straight-line segments.

### Hyperflagellation imposes significant metabolic costs

**Fig 5:**
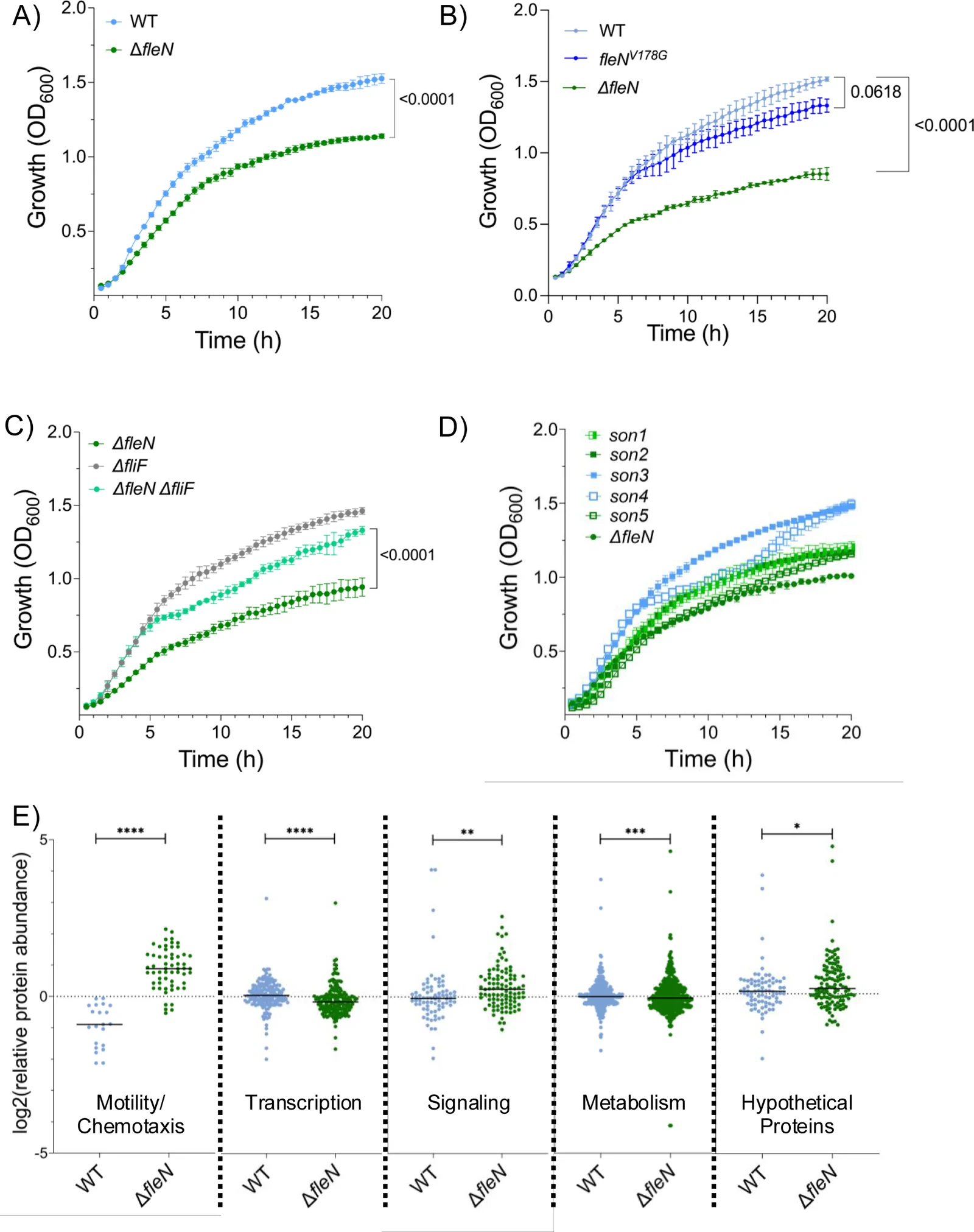
∆*fleN* mutant exhibits a growth defect compared to WT. (**A**) Growth curve of WT and ∆*fleN* cells, *n* = 3. (**B**) Growth curve of WT, ∆*fleN,* and *fleN^V178G^* strains, *n* = 3. (**C**) Growth curve of ∆*fliF*, ∆*fleN,* and ∆*fleN*∆*fliF* strains, *n* = 3. (**D**) Growth curve of ∆*fleN* suppressors, *son1–5*. For panels **A–C**, statistical significance was determined using the Mann-Whitney test. Effect size metric Hodges-Lehmann: (**A**) WT vs ∆*fleN =* −0.3523, (**B**) WT vs ∆*fleN =* −0.5523; ∆*fleN* vs *fleN^V178G^* = −0.1387; and (**C**) ∆*fleN* vs ∆*fleN*∆*fliF =* 0.3027. The results were considered not statistically significant when *P* > 0.05. Error bars represent the SD of three biological replicates. (**E**) Proteomics analysis of WT and ∆*fleN* strains. Statistical significance was determined using the Kolmogorov-Smirnov (KS) test. Effect size metric Kolmogorov-Smirnov D of WT vs ∆*fleN:* motility/chemotaxis = 0.9138, transcription = 0.2730, signaling = 0.2713, metabolism = 0.1692, and hypothetical proteins = 0.2292. *P < 0.05, **P < 0.01, ***P < 0.001, ****P < 0.0001.

To determine the broader physiological consequences of hyperflagellation, we performed comparative growth analysis and discovered that ∆*fleN* cells exhibited significantly slower growth kinetics compared to WT (Fig. 5A). While WT cultures reached stationary phase (OD_600_ = 1.5) within 20 h, ∆*fleN* cultures achieved only OD_600_ = 1.0 in the same timeframe (Fig. 5A). Serial dilution experiments confirmed equal viability between strains, but ∆*fleN* colonies remained smaller after extended incubation, indicating sustained growth defects (Fig. S6A). The growth defect in ∆*fleN* cells can be restored to wild-type levels in a FleN concentration-dependent manner (Fig. S2C). Furthermore, cell morphological analysis during exponential phase revealed that ∆*fleN* cells displayed heterogeneous sizes, with subpopulations of cells either larger or smaller than WT (Fig. S6B). This size heterogeneity correlates with the observed growth rate reduction and suggests that hyperflagellation affects fundamental cellular processes beyond flagellar assembly and motility. Since FliF is required early in flagellar assembly, *fliF* deletion blocks production of all downstream flagellar components, substantially reducing the total metabolic investment in flagellar biosynthesis. To test whether growth defects resulted from the metabolic burden of flagellar overproduction, we constructed deletion strains removing *fliF* (encoding a basal body component, 64.0 kDa) from WT and ∆*fleN* backgrounds. Absence of FliF significantly improved growth compared to ∆*fleN* alone (*P* < 0.0001), although not to WT levels (Fig. 5C). In our experimental conditions, the hyperswimmer and hyperflagellated *fleN*^V178G^ mutant showed no statistically significant growth difference compared to WT (Fig. 5B). We note that prior hyperswarming studies have reported a minor but statistically significant growth cost for *fleN*^V178G^ (14); the difference from our data set may reflect differences in growth conditions, and we therefore present this comparison without asserting equivalence between the two settings. Nonetheless, the growth dynamics of suppressors of ∆*fleN* correlate with the number of flagella per cell: single flagellated suppressors (*son3* and *son4*) recover growth dynamics similar to WT, while hyperflagellated suppressors (*son1, son2,* and *son5*) exhibit growth dynamics that range in between WT and ∆*fleN* growth curves (Fig. 5D). The partial growth restoration confirms that flagellar overproduction contributes significantly to the fitness costs observed in ∆*fleN* cells, although additional factors beyond simple metabolic burden are likely involved.

To comprehensively assess the molecular consequences of hyperflagellation, we performed quantitative proteomics using the diDO-IPTL method (70) to compare protein abundances between WT and ∆*fleN* cells during exponential growth (Fig. 5E). We classified differentially abundant proteins into functional categories based on Gene Ontology terms: motility and chemotaxis, metabolism and biosynthesis, signaling, transcription, cell division and DNA replication, stress response and protection, and unclassified proteins. As expected, proteins in the motility and chemotaxis category showed significant overabundance in ∆*fleN* cells, including core flagellar structural components (FlgE, FliF, FliC, and FliM) and motor proteins (MotC), confirming the hyperflagellation phenotype at the molecular level (Fig. 5E).

However, extensive changes occurred across all other functional categories, revealing that *fleN* deletion triggered broad cellular reprogramming beyond flagellar assembly. Several metabolic enzymes increased significantly, including cysteine synthase A (PA14_29110, upregulated ~8-fold), aconitate hydratase (PA14_44290, upregulated ~13-fold), and acyl carrier protein AcpP (PA14_25670, upregulated >3-fold), while cytochrome c4 (PA14_72460) showed over 4-fold reduction. Signaling pathway proteins demonstrated coordinated upregulation, particularly phenazine biosynthesis enzymes (PhzE, PhzB, PhzC, and PhzF) and two-component regulatory systems (CheY and FleR). Furthermore, proteins such as bacterioferritin (PA14_18670, upregulated 4.8-fold) and catalase (PA14_09150, upregulated ~4-fold) were more abundant in the ∆*fleN* mutant compared to WT, suggesting the activation of systems linked to stress responses. Transcriptional regulatory proteins displayed particularly striking alterations: while transcriptional machinery was downregulated overall in the ∆*fleN* mutant compared to WT, among the most abundant proteins in ∆*fleN* cells were PA14_48770 (putative transcriptional regulator from the LysR family), transcription termination factor Rho (PA14_69190), the stationary phase sigma factor RpoS (PA14_17480), and the flagellar sigma factor FliA (PA14_45630). This pattern suggests that hyperflagellation activates alternative transcriptional programs, possibly as stress responses to the altered cellular state. In addition, several hypothetical proteins including PA14_03350, PA14_44080, PA14_50570, PA14_58330, and PA14_69090 were greater than 5-fold more abundant in the ∆*fleN* mutant compared to WT. Taken together, these global proteomic changes reflect altered membrane composition requirements for supporting multiple flagellar assemblies or metabolic adaptations to the energetic costs of hyperflagellation.

### Hyperflagellation severely reduces competitive fitness, pathogenic capacity, and biofilm formation

**Fig 6:**
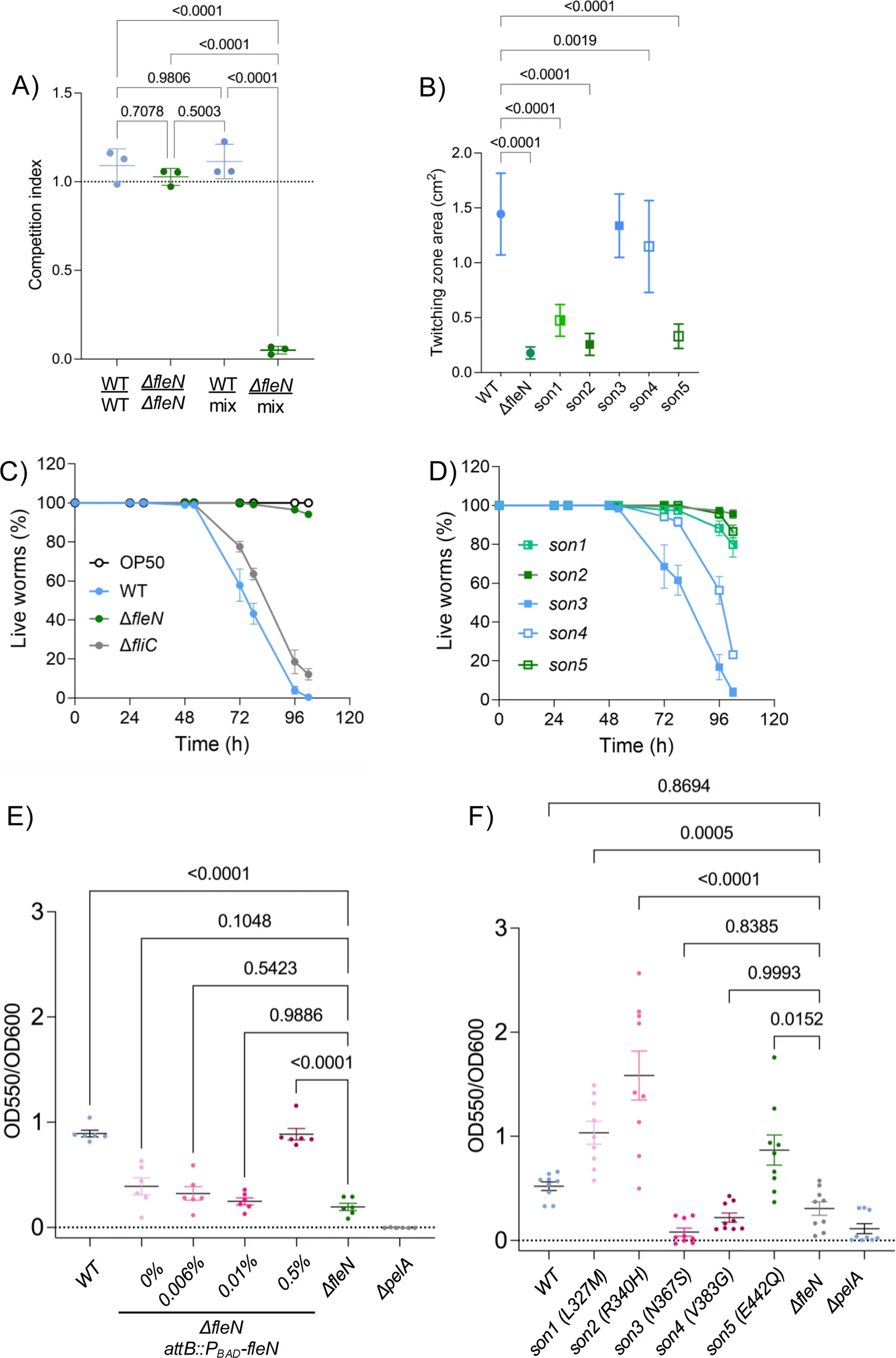
Hyperflagellated strains exhibit competitive disadvantage and decreased twitching and virulence. (**A**) Competition between WT and ∆*fleN* cells in shaken cultures. Competition index (CI) is the ratio between the number of cells of each strain at the end of the assay relative to the number of cells of each strain at the beginning of the assay, *n* = 3. CI ~1 = no difference, CI < 1 = mutant cells decrease, CI > 1 = mutant cells increase. (**B**) Twitching motility of WT, ∆*fleN and* ∆*fleN* suppressor strains. For panels** A** and **B**, error bars represent the SD of three biological replicates. (**C and D**) Worm slow-killing assay where *Caenorhabditis elegans* were applied to lawns of *E. coli* OP50 and the indicated WT and mutant *P. aeruginosa* strains. Error bars represent SEM of three independent experiments. (**E and F**) Biofilm crystal violet staining assays for WT and indicated mutant strains. Error bars represent SEM of (**E**) six and (**F**) nine biological replicates from at least two independent experiments. Only pairwise comparisons against ∆*fleN* are denoted. Statistical significance for panels** A**, **B**, **E**, and **F** was determined using one-way ANOVA, followed by a Tukey’s post-hoc multiple comparisons test (Panel **A**: R^2^ = [0.9828]; Panel **B**: *R*^2^ =[0.8390]; Panel **E**: *R*^2^ = [0.8921]; Panel** F**: *R*^2^ = [0.7126]). The results were considered not statistically significant when *P* > 0.05.

To assess the ecological consequences of hyperflagellation, we performed direct competition experiments between WT and ∆*fleN* cells in liquid culture. Starting with equal cell densities, WT cells achieved complete dominance within 17 h, reaching a >10:1 ratio in colony-forming units (Fig. 6A; Fig. S6C). This competitive disadvantage confirms that the growth defects observed in monoculture translate to severe fitness costs under competitive conditions. Furthermore, investigation of the consequences of hyperflagellation on type IV pili mediated twitching motility showed inverse correlation such that the ∆*fleN* mutant was severely impaired in twitching (Fig. 6B). Consistent with this, analysis of the five *son* strains revealed that suppressors that restored monotrichous flagellation (*son3* and *son4*) maintained WT level twitching, while those retaining hyperflagellation (*son1, son2,* and *son5*) showed severely reduced twitching despite their improved swimming ability (Fig. 6B). We conclude that hyperflagellation hinders twitching motility.

Next, we investigated pathogenic capacity using the *C. elegans* slow-killing assay, a well-established model for *P. aeruginosa* virulence (71–73). WT PA14 demonstrated full pathogenic potential, killing 100% of exposed worms within 96 h (Fig. 6C). In striking contrast, the ∆*fleN* mutant showed virtually no pathogenicity, with worm survival rates comparable to non-pathogenic *E. coli* OP50 control bacteria (Fig. 6C). To determine whether this virulence defect resulted from impaired motility or other consequences of FleN deletion, we tested a non-motile ∆*fliC* mutant lacking the flagellar filament entirely. Remarkably, non-flagellated cells retained complete pathogenic capacity, killing worms with kinetics identical to WT (Fig. 6C). This result demonstrates that flagellar motility or the flagellum itself is not required for PA14 pathogenesis in this model system, implying that hyperflagellation specifically, rather than loss of motility/flagella *per se*, impairs virulence mechanisms. Consistent with this, analysis of the five *fleQ* suppressor strains revealed a clear correlation between flagellar assembly phenotype and pathogenic capacity. Suppressors that restored monotrichous flagellation (*son3* and *son4*) maintained full virulence, while those retaining hyperflagellation (*son1, son2,* and *son5*) remained avirulent despite their improved swimming ability (Fig. 6D). We conclude that hyperflagellation, rather than the loss of motility, impairs pathogenic processes.

*P. aeruginosa’s* ability to form biofilms confers resistance against antibiotics and immune system evasion, allowing successful niche colonization (74). We tested whether hyperflagellation affects biofilm formation using 1% crystal violet (CV) staining (Fig. 6E and F). Hyperflagellated ∆*fleN* cells exhibited a severe biofilm formation defect compared to WT (Fig. 6E). Therefore, we hypothesized that ∆*fleN* cells would progressively recover biofilm formation ability in proportion to the level of *fleN* induction (∆*fleN attB::P_BAD_-fleN*). Our results revealed a striking dose-dependent dichotomy: while 0.01% arabinose fully restored swimming area to wild-type levels (Fig. 2A), biofilm formation remained impaired (Fig. 6E). Conversely, although 0.5% arabinose completely abolished swimming motility (Fig. 2A), this same concentration restored biofilm formation to wild-type levels (Fig. 6E). These findings demonstrate that FleN presence alone is insufficient for biofilm recovery; rather, a specific FleN concentration threshold is required. Moreover, these results underscore how a single FleN concentration can exert opposing effects on distinct biological functions.

We next assessed the biofilm formation capacity of the *son* strains (Fig. 6F). Consistent with the motility heterogeneity observed within each *son* strain, CV staining revealed heterogeneous biofilm formation among the hyperflagellated populations (*son1*, *son2*, and *son5*) (Fig. 6F). Notably, strains that had recovered wild-type flagellar number (Fig. 3D) and swimming area (Fig. 3B), specifically *son3* and son4, failed to form biofilms (Fig. 6F), revealing an unexpected disconnect between flagellar number restoration and biofilm formation. In conclusion, these results suggest that hyperflagellation does not abolish biofilm formation, that there is likely a different threshold of FleN concentration for recovering biofilm formation, and that the ability to form biofilms is not required for pathogenicity in *C. elegans*, as previously reported (75, 76).

## DISCUSSION

Bacterial flagellar systems illustrate how regulatory circuits balance phenotypic constraint with adaptive potential. Our findings reveal that the FleQ-FleN circuit governing flagellar number in *P. aeruginosa* acts not only as a developmental checkpoint but also as an evolutionarily plastic system capable of rapid reconfiguration under laboratory selection. Notably, the fitness-restoring *son* suppressors we identified maintain rather than eliminate the hyperflagellated state, challenging the assumption that multiflagellation is inherently maladaptive. Together, these observations suggest that flagellar number is a tunable parameter in bacterial life-history strategy.

### Suppressor genetics illuminates regulatory circuit architecture

The spectrum of mutations in *fleQ* provides structure-function insight complementary to prior biochemical studies. Although designed mutations in conserved catalytic motifs abolish FleQ ATPase activity entirely [41], our suppressor mutants behave genetically as partial, rather than complete, loss-of-function alleles. Residual transcription of the class II gene *fliF* indicates that FleQ retains transcriptional activity in these mutants, consistent with partial loss of function. Although *fliF* transcript levels in the *son3* mutant were lower than wild type, consistent with >50% of cells in that population lacking flagella, its flagellated fraction still reached wild-type spreading areas in soft agar. Four of five suppressors mapped to the AAA+ ATPase domain, consistent with the central role of ATP hydrolysis in σ54-dependent transcriptional activation [41]; the fifth mapped to the DNA-binding domain, suggesting that DNA binding, not just the predicted catalytic function, can be perturbed to rebalance the circuit (we note that we measured neither promoter occupancy nor ATPase activity directly). These observations align with structural data on FleQ-FleN complexes [42] showing large-scale conformational switching between motility and biofilm states, and our alleles may shift this equilibrium, reducing flagellar gene expression enough to ameliorate fitness costs while maintaining elevated flagellar number.

The dissociation between flagellar pattern, swimming efficiency, and biofilm formation in some suppressors (e.g., *son4* with WT-like flagellation but minimal swimming gain and no biofilm, while *son1* hyperflagellated yet swimming and forming biofilm efficiently) indicates that factors beyond flagellar number and arrangement, such as coordinated rotation, chemotaxis integration, metabolic efficiency, or FleQ-independent circuits, influence motility. FleQ AAA+ mutations likely affect more than σ^54^-dependent transcription, as FleQ can act independently of FleN, ATPase activity, σ^54^ (42, 43, 77), and c-di-GMP binding to the AAA+ domain modulates its ATPase activity, oligomerization, conformation, and DNA-binding affinity, regulating targets beyond the flagellar and biofilm regulons (47, 49, 50). We did not measure intracellular c-di-GMP; we therefore raise as a hypothesis that the diversity seen in Δ*fleN* suppressors and across FleN titration may involve altered c-di-GMP signaling through this domain.

### Phenotypic heterogeneity as an adaptive strategy

A striking feature of our suppressor strains is their single-cell heterogeneity: subpopulations achieve wild-type swimming despite bearing multiple flagella, while others exhibit impaired motility. Phenotypic heterogeneity within clonal populations represents an adaptive bet-hedging strategy (78–80), potentially enabling populations to simultaneously exploit environments favoring rapid swimming and those favoring reduced motility (e.g., biofilm initiation). The FleQ-FleN circuit, through its integration of c-di-GMP signaling (43, 50), is well-positioned to generate such diversity. Our data suggest that circuit perturbation followed by compensatory evolution expands the population’s available phenotypic repertoire.

On the technical side, we have introduced the use of digital in-line holography (DIH) to visualize the trajectories of *P. aeruginosa* cells in three dimensions. Compared to other commonly used particle tracking techniques, DIH has the advantages of straightforward implementation and suitability to track cells precisely even in a high-density culture (81). Using DIH, we observed three broad categories of trajectory for individual *P. aeruginosa* cells: straight (most common in WT), meandering (observed with bipolar hyperflagellated cells), and helical (observed in ∆*fleN* and suppressor mutant populations). More in-depth analysis of the trajectories in the future, potentially separating the different subpopulations, will lead to more biophysical insights on the effect of multiflagellation on the persistence and robustness of the flagella-based motility. The relative sizes of the subpopulations displaying these motility patterns are affected by the FleN-FleQ regulatory axis, creating opportunities for the most motile subpopulations to be selected for by environmental conditions. The increased diversity in swimming behaviors for hyperflagellated strains may lead to the evolutionary potential for the hyperflagellated phenotype to be selected, as is naturally seen in several other *Pseudomonas* species.

### Consequences of chronic hyperflagellation

Our proteomics results indicate that hyperflagellation is accompanied by reprogramming beyond the flagellar regulon, affecting central carbon metabolism, oxidative-stress responses, and two-component signaling. This may reflect both direct consequences of FleQ hyperactivity as a global regulator (42) and the metabolic burden of excess flagellar biosynthesis—a substantial cost, given that flagellar proteins can consume 2%–8% of the *E. coli* proteome (18), with relative construction costs spanning nearly two orders of magnitude across bacterial species (19), and that flagellar deletion improves oxidative-stress tolerance in *P. putida* (82). These changes carry ecological implications: downregulated virulence pathways may contribute to the attenuated pathogenicity we observe, while upregulated stress responses might aid survival where improved tolerance offsets slower growth, that is, trade-offs meriting study in ecologically relevant contexts.

### Evolutionary accessibility of alternative flagellar configurations

Wild-type *P. aeruginosa* can evolve toward hyperflagellation under viscous laboratory selection, paralleling swarming experimental evolution in which *fleN* point mutations arise reproducibly (14, 83). Li et al. (84) found *fleN* mutations reaching 49% frequency during experimental evolution of *Pseudomonas bijieensis* in the wheat rhizosphere, increasing flagellar number, motility, and colonization, indicating that the trajectories we document in the laboratory can also arise in a plant-associated experimental-evolution setting. Together, these findings establish that the path from monoflagellation to multiflagellation is evolutionarily accessible under a range of selection pressures, including ecologically relevant ones. The FlhF/FlhG (FleN) system is conserved across polar flagellates including *Vibrio, Campylobacter, Helicobacter*, and *Shewanella* (64, 85–89), with FlhG/FleN acting as a negative regulator whose loss yields hyperflagellation; its phylogenetic breadth and repeated perturbation under selection suggest that numerical control of flagellation is a conserved axis of variation with adaptive potential.

### The FleQ-FleN circuit as a candidate evolutionary capacitor

We interpret these laboratory observations through the framework of evolutionary capacitance, that is, a system that constrains phenotypic variation under standard conditions while harboring latent potential released under selection (90, 91), as in Hsp90-mediated buffering of cryptic variation (92) and prion-based switching (93). We offer this as an interpretive framework, not a demonstrated property: unlike those examples, we have not shown that the circuit buffers and releases standing genetic variation, and every allele here was recovered under laboratory selection and carries pleiotropic costs (Fig. 5 and 6). With that caveat, several features are consistent with capacitor-like behavior. The circuit normally restricts flagellar number to one polar flagellum despite the capacity for more; perturbing this constraint reveals previously suppressed variation in flagellar number, swimming, and downstream states; a subset proves adaptive under our selection, as suppressors restore fitness while retaining alternative configurations; and the transition is reversible at the population level. This is consistent with viewing FleQ-FleN as a c-di-GMP-responsive node governing motile-versus-sessile commitment, where minor perturbations tune the threshold and stronger ones (∆*fleN* or constitutive c-di-GMP elevation) lock the circuit into hyperflagellation or biofilm states; either extreme eliminates the transitional flexibility that is advantageous where selective pressures alternate unpredictably. Whether this framework describes natural evolution remains untested and would require population-genetic analysis of natural isolates. With those limits stated, the framework may still help explain adaptation in fluctuating environments, including viscous or structured niches such as mucus, root surfaces, or biofilm matrices that may favor increased thrust, and single-mutation access to multiflagellated states could let populations exploit latent biosynthetic capacity rather than evolve new machinery.

### Clinical and ecological implications

Hyperflagellation imposes fitness costs beyond impaired motility: the reduced growth, altered morphology, and compromised pathogenicity of ∆*fleN* highlight the consequences of dysregulated FleQ-FleN axis. Restored growth in ∆*fleN*∆*fliF* indicates that the burden stems mainly from basal-body overproduction rather than filament proteins, consistent with the hierarchy of flagellar gene expression (41, 94). The differential pathogenicity between monotrichous and hyperflagellated strains adds another dimension to this fitness equation. In the *C. elegans* model, the loss of virulence in hyperflagellated strains, despite flagellar motility being dispensable for pathogenesis (95, 96), suggests effects beyond motility, perhaps altered immune recognition or impaired virulence-factor secretion. Importantly, although these *fleQ* alleles were derived by laboratory selection in our study, *fleQ* is also a recurrent target of natural selection: clinical isolates harbor *fleQ* mutations (e.g., PA103, acute pneumonia (15)), and the sputum isolate PA108 carries our *son4* allele, FleQ^V383G^ (97), although establishing their fitness consequences in clinical or environmental settings is beyond this study.

We note that while unipolar hyperflagellation can confer increased motility, we find a precipitous decrease in motility in strains with >4 flagella at one pole. This finding resonates with ecological studies showing that bacteria can adapt their motility systems to diverse habitats (20, 98). Similarly, *P. fluorescens* engineered to lack flagellar motility via deletion of *fleQ* consistently regained swimming ability by evolving the NtrBC regulatory axis to function as an activator of flagellar gene transcription (99, 100). The rarity of hyperflagellation from the wild-type background in our setup, versus the frequent ∆*fleN* suppressors, suggests transitions from monotrichous to alternative flagellar patterns are tightly controlled and arise only under strong selection, reflecting the favorable cost-benefit ratio of monotrichous flagellation across *P. aeruginosa*’s diverse habitats. Consistent with a broader ecological range, synteny analysis (Fig. S7) shows high conservation of flagellar gene clusters between monotrichous (*P. aeruginosa*) and lophotrichous (*P. putida* and *P. syringae*) Pseudomonas, which share phyllosphere and rhizosphere niches (32, 33, 62, 66, 101–103). Within the rhizosphere, Pseudomonas communities express variable flagellar gene levels (104), echoing our patterning heterogeneity. FleQ and FleN are highly conserved, FliC less so (Figs. S8 to S10), possibly optimizing filament coordination for rhizosphere viscosity. In contrast to these motility-restoring routes, loss-of-function mutations in the Wsp surface-sensing pathway elevate c-di-GMP to drive the sessile, hyper-biofilm state, a recurrent compensatory trajectory in chronic infection and *in vitro* biofilm evolution (105); the same motile-versus-sessile balance can thus be retuned from either direction, each with its own trade-offs. Indeed, in an engineered *fleN^V178G^* ∆*wspF* strain, which is immotile and biofilm-competent through elevated c-di-GMP, selection for regained swarming recurrently produced Wsp-disabling mutations, including in *wspA*, retuning the balance through the Wsp/c-di-GMP axis rather than, as in our suppressors, through FleQ (105).

### Conclusions and future directions

Our work characterizes the FleQ-FleN circuit as an evolvable regulatory node that tunes flagellar number. Our laboratory-evolution approach gives controlled insight, but natural and clinical pressures in rhizosphere, marine, or airway niches likely add complexity; causal links between the proteomic changes and fitness and the mechanistic basis of improved multiflagellated swimming remain open. Future work should address the structural consequences of suppressor mutations for the FleQ-FleN interaction, direct c-di-GMP measurement across the allelic series, flagellar-coordination mechanisms, and the origins of heterogeneity (stochastic expression, asymmetric division, or other processes).

## MATERIALS AND METHODS

### Strains and growth conditions

*P. aeruginosa* UCBPP-PA14 strain was grown in lysogeny broth (LB) (10 g tryptone, 5 g yeast extract, and 5 g NaCl per L), in 1% tryptone broth (TB) (10 g tryptone per L), and on LB plates fortified with 1.5% Bacto agar at 37°C (106). When appropriate, antibiotics were included at the following concentrations: 400 µg/mL carbenicillin, 50 µg/mL gentamicin, 100 µg/mL irgasan, and trimethoprim 500 µg/mL. For the CFU quantification, overnight cultures were diluted to 1:100 into fresh LB and grown over the day until reaching exponential phase (OD_600_ = 0.5). Cultures were again diluted to reach a new concentration of OD_600_ of 0.05 and grown for 20 h. After 20 h, 10-fold dilutions were plated on LB plates and grown at 37°C overnight. Counting of colonies was done manually. For the growth curves, overnight cultures of bacteria were diluted to an OD_600_ of 0.05 in 250 µL of fresh LB in replicates using a 96-well plate. Readings were taken every 30 min for 20 h at 37°C with constant shaking. The optical density at 600 nm was measured using a Synergy Neo2 microplate reader (Agilent BioTek).

### Strain construction

Strains and plasmids were constructed as described previously (106, 107). To construct marker-less in-frame chromosomal deletions in *P. aeruginosa*, DNA fragments flanking the gene of interest were amplified, assembled by the Gibson method, and cloned into pEXG2 (108). The resulting plasmids were used to transform *E. coli* SM10λpir and, subsequently, mobilized into *P. aeruginosa* PA14 via biparental mating. Exconjugants were selected on LB containing gentamicin and irgasan, followed by the recovery of deletion mutants on LB medium containing 5% sucrose. Candidate mutants were confirmed by PCR. For the construction of *son* mutants, PA14 genomic DNA from the isolated Δ*fleN* suppressors was used as a template; ~1,000 bp upstream and downstream of *fleQ* (including *fleQ*) was amplified and cloned into pEXG2. The resulting plasmids were used to transform *E. coli SM10λpir* and, subsequently, mobilized into *P. aeruginosa* PA14 Δ*fleQ* or Δ*fleN*Δ*fleQ*, respectively.

The complementation strain of *fleN* was cloned into pTJ1-mini-Tn7T-Tp plasmid (106). The resulting plasmids were transformed into *E. coli* DH5α and mobilized into *P. aeruginosa* PA14 via quad-parental mating using as helper strains DH5α/pTNS3 and DH5α/pRK2013 to facilitate Tn7 transposition at the attB site as described here (106). Insertion of the Tn7 transposon region was confirmed by PCR followed by Sanger sequencing.

### Complementation of *fleN*

The L(+)-arabinose 99% (AC365180250) was dissolved in Milli-Q-water and filter sterilized before using it. Cells ablated from *fleN* and carrying a *fleN* insertion under the P_BAD_ were grown in liquid LB overnight without arabinose. For the growth curves, microscopy and swimming assays, arabinose was added at the indicated concentration at the beginning of each experiment.

### Flagella and membrane staining

*Pseudomonas* cells’ flagellin (*fliC*) gene was exchanged with a version of *fliC* (*fliC^T394C^*) able to react with Alexa Fluor 488 C5 Maleimide from Thermo Fisher (cat. no. A10254) (59). The new strain became the wild-type background used to build all the mutant strains used in this paper. Overnight cultures were diluted 1:100 in 3 mL LB media and then grown over the day at 37°C until reaching the desired OD_600_ (between 0.25 and 1.5). The ends of the pipette tips were cut off consistently for all the staining procedures to minimize flagellar damage from shear; 500 µL of bacterial culture was centrifuged for 1 min at 15,000 rpm, the supernatant was discarded, and the pellet was mixed with Alexa Fluor in 50 µL of 1× PBS buffer (1:10 dilution). Cells were incubated in the dark for 3 min, and then 1 mL of 1× PBS was added gently to wash. Cells were then centrifuged for 1 min at 15,000 rpm, and the supernatant was discarded. For the membrane staining, similar steps to the flagella staining were used, but using the membrane dye FM464 instead of Alexa Fluor. Cells were incubated in 50 µL (10 µg/mL) of FM464 (Invitrogen T13320) for 2 min and then washed with 1× PBS. After centrifugation and decanting the supernatant, the pellet was gently resuspended in ~50 µL of 1× PBS buffer; 10 µL of the sample was placed into a microscopy slide (previously cleaned with 70% isopropanol) and pressed down with a coverslip (previously soaked in poly-L-lysine to immobilize the cells). All microscopy images were taken using the inverted confocal microscope Leica Stellaris 5. Images were then processed using Fiji. Quantification of the number and polarity of flagella was done manually, and values were graphed using GraphPad.

### Holographic tracking of cell trajectories in three dimensions

To observe the three-dimensional trajectories of bacteria swimming in liquid media, overnight culture was regrown in LB, cultured at 37°C with shaking for 3.5 h, until OD_600_ reached approximately 0.8. This was diluted in fresh LB to a concentration with an OD_600_ of 0.002. Samples were prepared by pipetting 20 µL of diluted culture into silicone gaskets (Grace Biolabs) adhered to a cleaned glass coverslip. Digital in-line holography was accomplished with a custom laser illumination setup similar to that described before (109) on a Nikon Ti-E using a 4× objective with 1.5× intermediate magnification, yielding videos at a resolution of 0.559 µm per pixel in a field size of 0.33 mm^2^. Raw holograms were captured for 15 s at 120 frames per second on a Teledyne FLIR camera (BFS-U3-32S4M-C) illuminated with a 405 nm laser (Thorlabs) at approximately 5 mW, collimated to a beam waist diameter of 5.3 mm. After background subtraction, holograms were processed into X, Y, and Z coordinates for the cells using the Regularized Inverse Holographic Volumetric Reconstruction (RIHVR) algorithm (109) using a computer with a GeForce RTX 4090 GPU. The Python package trackpy (110) was used to link the trajectories for further analysis with custom Python code.

### Dual-color flagellar observation assay

To fluorescently label flagella, we used a previously described method (59) where each cysteine-mutated strain (*fliC^T394C^*) was inoculated from frozen stock into LB and cultured overnight at 30°C with shaking. Overnight culture was regrown in LB, cultured at 37°C with shaking for 3.5 h, until OD_600_ reached 0.8. Then, the regrown culture was centrifuged at 4,000 rcf for 4 min, and the supernatant was removed and replaced with Alexa Fluor C5-maleimide (Thermo Fisher) at a concentration of 25 µg/mL diluted in LB. Cells were resuspended by gentle pipetting. This solution was incubated at room temperature for 10 min before being washed three times by centrifuging cells as before, replacing supernatant with fresh LB, and gently pipette mixing the cells to resuspend. All cell transfers were accomplished with pipette tips that had been pre-cut to widen the opening and prevent flagellar damage due to shear.

Cells were transferred into 10-µm-tall PDMS microchannels with glass bottoms for imaging using EPI-illumination on a Nikon Ti2-E. Simultaneous dual-color video was achieved with a Cairn OptoSplit II placed at the end of the light path just before the EMCCD, an Andor Ixon Life 888.

### Three-dimensional single-cell trajectories analysis

Three-dimensional single-cell trajectories were analyzed in Python3 using a custom Jupyter notebook script built with packages utilizing NumPy (1.24.3) (111), Pandas (2.0.3) (112), Matplotlib (3.7.1) (113), Plotly (5.22.0) (114), and SciPy (1.11.1) (115). The custom scripts used to generate Fig. 4E and F are available on GitHub (https://github.com/strocha1/3D-trajectory-analysis). Trajectory tables were imported from CSV files for each condition (Ex. WT or ∆*fleN*), and only measured (non-interpolated) positions were retained. Data were grouped by particle identifier and ordered by frame number, where velocity (v) was available; low-motility frames were removed using a minimum velocity threshold of 1.0 µm/s, and trajectories with fewer than three remaining frames were excluded from downstream calculations.

All *x*/*y*/*z* coordinates were denoised independently using a Savitzky-Golay filter (window length = 5 frames, polyorder = 1). Stepwise 3D displacements were calculated as the Euclidean distance between successive smoothed positions. Distance per trajectory was defined as the sum of all consecutive three-dimensional displacements across the entire track for a given particle (giving cumulative path length, not net displacement). Pixels were converted using a spatial scaling factor of 0.559 µm per pixel, and path lengths were divided by trajectory duration to give average trajectory velocity, given a frame rate of 120 frames per second.

For quality control via manual visualization, up to 10 trajectories per condition were randomly sampled and exported as interactive 3D overlays to compare the raw and smoothed tracks, along with per-trajectory velocity vs time plots. Condition-level distributions were summarized using histograms, kernel density estimates (PDF), and empirical cumulative distribution functions (CDF) (Fig. 4E and F).

### Swimming motility assay

To an autoclaved solution of 0.3% agar medium, 200 mL of 5× M8 salts were added, together with 5 mL of 0.2% casamino acids and 40% glucose, respectively, plus 1 mL of MgSO_4_. When appropriate, antibiotics or induction molecules were added at this point. Plates were stacked into piles of 3 and allowed to solidify at room temperature for 4–5 h. Overnight cultures (or grown over the day, when specified) were inoculated into the agar by inserting a pipette tip (without reaching the end of the plate) slightly soaked with bacteria. Plates were placed inside containers with soaked paper towels in the base of the container and covering the plates. The plates were placed inside containers with soaked paper towels in the base and top of the container and incubated at 37°C with a water bucket inside the incubator to maintain humidity. Unless otherwise indicated, plates were imaged after 21 h using Amersham ImageQuant 800. The swimming area was measured using Fiji and plotted using GraphPad.

### Twitching motility assay

Overnight cultures were inoculated into the flat bottom of a 1.5% agar plate (20 mL each) using skewers. The skewer’s tip was submerged into the overnight cultures, and then the tip was pushed through the 20 mL of the solidified agar. Incubation of the plates was done for 48 h at 37°C. To stain the twitching area, first a spatula was used to trace along the edge of the plate and lever the agar out of the plate. Next, a thin layer of 0.1% crystal violet was poured on top of the twitching areas (just enough to cover the surface of the plate) and incubated for 20 min at room temperature. When ready, the crystal violet was discarded into a waste beaker, and then, the plate was submerged inside a bowl of clean water to wash off the excess crystal violet. After letting the plate dry (~20 min), the colorimetric images were collected using the Amersham ImageQuant 800 (Cytiva) imager. Further processing (quantification of twitching area) of the images was done in Fiji with manual tracing of the area. Values were graphed using GraphPad.

### Crystal violet staining of biofilms

*P. aeruginosa* was cultured overnight in 200 μL LB in a BioTek Synergy Neo2 microplate reader at 37°C under shaking conditions as described previously (116). The OD_600_ of the culture was taken by the plate reader to calculate the amount of culture needed to inoculate 200 μL 1% Tryptone broth so that the final OD_600_ is 0.005. Biofilms were developed for 72 h in a stationary 96-well polystyrene plate at 25°C in dark conditions. After 72 h, OD_600_ measurement was taken, and the supernatant was discarded. The cultures were washed vigorously with tap water and were left to dry for an hour. The biofilms were then stained with 250 μL 0.1% crystal violet solution for half an hour. The excess solution was poured out; the samples were washed thrice with tap water and were left to dry overnight. For elution, 250 μL of 33% glacial acetic acid solution was used in each well, and the samples were left to rest for an hour. The crystal violet stain was quantified at OD_550_ using the microplate reader.

### Western blot assays

PA14 strains were grown overnight at 37°C in LB medium. The next day, the overnight cultures were diluted to 1:100 in fresh LB and then grown over the day at 37°C with shaking until reaching an OD_600_ of 0.5; 1 mL of cell culture was harvested and resuspended in 200 μL of 2× loading buffer (50% 2× Tris buffer [10 mM Tris pH 6.8, 1.5 mM bromophenol blue], 45% 2× sample buffer [125 mM Tris pH 6.8, 20% glycerol, 210 mM SDS, 10% BME], and 5% BME). The samples were heated for 10 min at 95°C; 10 μL samples were loaded onto 12% SDS-PAGE gels and were subjected to electrophoresis. Proteins were transferred to nitrocellulose membranes overnight (10 V for 16 h) at 4°C on a rocking platform. The next day, the membrane was blocked with 5% skim milk in TBS at room temperature for 1 h. Following, an incubation with primary antibodies anti-FleQ and anti-RNAP (Abcam, Inc., 469 Cambridge, UK) at 1:2,500 and 1:10,000 dilution, respectively, in 5% skim milk in TBS was performed for 2 h at room temperature on a rocking platform. Membranes were washed three times with TBS-Tween 20 at room temperature for 10 min on a rocking platform. Incubation with the secondary antibody anti-rabbit IgG-peroxidase produced in goat (Sigma-Aldrich) was followed for 1 h at room temperature. The membranes were washed three times again with TBS-Tween 20 for 10 min and subsequently developed with a SuperSignal West Femto Kit (Thermo Scientific) and captured with an Amersham ImageQuant 800 (Cytiva) imager. Quantification of the western blots was performed with Image Lab 6.1 software (Bio-Rad).

### Fluorescent microscopy

Each strain of bacteria was streaked out on LB plates and left at 37°C for 16 h, and overnight cultures of single colonies were set. The next day, overnight cultures were diluted at 1:100 dilution in fresh LB and grown over the day until reaching the desired OD_600_. Unless indicated, 500 μL of the overnight culture was centrifuged for 2 min at 15,000 rpm and prepared for flagella staining; 5 μL of the samples were loaded onto a microscope slide and covered with a 1 mm poly-L-lysine coated coverslip. Micrographs were acquired at 63X magnification with immersion oil in a Stellaris 5 (Leica) microscope and processed using Fiji.

### Nutrient competition

Overnight cultures of bacteria were diluted (1:100) in fresh LB and then grown overnight until reaching an OD_600_ of 0.5. To prepare the cells for microscopy, 1 mL of cells of each strain was centrifuged for 2 min at 15,000 rpm, and the resulting pellet was resuspended in 500 μL of 1× PBS; 5 μL of sample was pipetted onto each microscopy slide. For the competition experiment, in 3 mL of LB, 5 μL of WT and mutant cells (at OD_600_ = 0.5) were mixed in a 1:1 ratio. Cultures were incubated while shaking for 18 h at 37°C. After the 18 h incubation, cultures were diluted to reach an OD_600_ of 0.5 and processed for fluorescence microscopy as previously described. To quantify the cells, 10 areas of interest (30 × 30 µm) per sample, per replica were analyzed using Fiji and manually counted. For the competition index, the number of cells per strain at the end of the experiment (output) was compared to the number of cells of the same strain at the beginning of the experiment (input). Regarding the mixed cultures, the number of cells per strain within the mixed culture at the end of the experiment (output) was compared to the number of cells of the same strain at the beginning of the experiment (input).

### Quantitative real-time PCR (qPCR) analysis

Overnight cultures of bacteria were diluted (1:100) in fresh LB and then grown over the day until reaching an OD_600_ of 0.5, and 1,000 µL of cell culture was pelleted for RNA extraction. Pellets were resuspended in TRIzol (200 µL), and total RNA was isolated using the Direct-zol RNA Miniprep Kit (Zymo Research) according to the manufacturer’s instructions. RNA (0.5 µg) was reverse-transcribed into cDNA using the PrimeScript RT Reagent Kit with gDNA Eraser (Takara Bio, San Jose, CA, USA). Quantitative real-time PCR (qPCR) was performed using the Applied Biosystems PowerTrack SYBR Green Master Mix on a Bio-Rad C1000 Touch Thermal Cycler. qPCR data were analyzed using the comparative Cq (ΔΔCq) method as described previously (106). Cq values of the *fliF* gene were normalized to the geometric mean of the reference gene *rpoD* to obtain ΔCq values. Relative expression levels were calculated as 2^−ΔΔCq^.

### Quantitative proteomics analysis

*P. aeruginosa* cell pellets were extracted by heating and vortexing at 95°C for 20 min in a reducing and denaturing buffer. SDS (1%)/Tris (200 mM, pH 8.0)/DTT (10 mM) and cysteine thiols were alkylated with 40 mM iodoacetamide. Proteins were then purified by a modified eFASP (enhanced filter-aided sample preparation) protocol (117), using Sartorius Vivacon 500 concentrators with a 30 kDa nominal cutoff. Proteins were digested with MS-grade trypsin (37°C overnight), and peptides were eluted from the concentrator and dried via vacuum centrifugation. Peptides were then isotopically labeled at N- and C-termini using the diDO-IPTL methodology (70) for quantitative proteomic analysis. Briefly, C-termini were labeled with oxygen-16 or -18 by an enzymatic exchange in isotopic water of a >98 atom % enrichment, and N-termini were labeled with un- or dideuterated formaldehyde via reductive alkylation using sodium cyanoborohydride. Peptide extracts from each sample were split, and aliquots were labeled separately with CD_2_O/H_2_^16^O and CH_2_O/H_2_^18^O; the latter were pooled to serve as a common internal standard for quantification. Aliquots of the ^16^O-labeled peptides and ^18^O-labeled internal standard were mixed 1:1 (vol/vol) and analyzed by LC-MS for protein expression quantification.

The peptide samples were then separated on a monolithic capillary C18 column (GL Sciences Monocap Ultra, 100 μm I.D. ×200 cm length) with a water-acetonitrile and 0.1% formic acid gradient (2%-50% ACN over 180 min) at 360 nL/min^−1^ using a Dionex Ultimate 3000 LC system with nanoelectrospray ionization (Proxeon Nanospray Flex source) for LC-MS analysis. Mass spectra were collected on an Orbitrap Elite mass spectrometer (Thermo) operating in data-dependent acquisition mode, with one high-resolution (120,000 *m*/Δ*m*) MS1 parent ion full scan triggering 15 rapid-mode MS2 CID fragment ion scans of selected precursors. Proteomic mass spectral data were analyzed using MorpheusFromAnotherPlace (MFAP) (70), with precursor and product ion mass tolerances set to 20 ppm and 0.6 Da, respectively. Static cysteine carbamidomethylation and variable methionine oxidation, N-terminal (d4)-dimethylation, and C-terminal ^18^O_2_ were included as modifications. The false discovery rate for peptide-spectrum matches was controlled by target-decoy searching to <0.5%. Protein-level relative abundances and standard errors were calculated in R using the Arm postprocessing scripts for diDO-IPTL data (70).

### Slow-killing worm assay

WT N2 worms (*C. elegans*) were propagated on *E. coli* OP50 lawns on Nematode Growth Media (NGM) plates. Gravid adults were collected and bleached to release the eggs as per protocol (118) and incubated at room temperature for 24 h to hatch. Approximately 40 h before the assay, 200–300 hatched L1 worms were plated onto NGM-OP50 plates and kept at 20°C to mature to the L4 stage. In parallel, 2 days before the assay, 10 μL of day cultures were inoculated onto the Slow Killing (SK) plates supplemented with 50 μg/mL 5-fluoro-2′deoxyuridine (FUDR) and incubated first at 37°C for 24 h and then at 20°C for an additional 24 h. L4 worms were washed with 5 mL M9 buffer and inoculated on SK plates. Scoring worms started within 24 h and for a duration of 4 days.

## Data Availability

Proteomic mass spectral data are available via the MassIVE repository (massive.ucsd.edu) under accession MSV000098684 (ftp://massive-ftp.ucsd.edu/v10/MSV000098684/). All other relevant data are provided within the article and the supplemental information.

## SUPPLEMENTAL MATERIAL

Supplemental material mbio.00360-26-s0001.pdf Supplemental figures, video legends, and tables

Video S1 mbio.00360-26-s0002.avi Flagellar wrapping affects bipolar multiflagellated motility.

Video S2 mbio.00360-26-s0003.avi Hyperflagellation can enhance swimming speed.

ASM does not own the copyrights to Supplemental Material that may be linked to, or accessed through, an article. The authors have granted ASM a non-exclusive, world-wide license to publish the Supplemental Material files. Please contact the corresponding author directly for reuse.
