# Supplementary material for: The FleQ-FleN circuit balances flagellar number against fitness in Pseudomonas aeruginosa
Source: mBio. 2026 Aug 5;17(9):e00360-26. doi: 10.1128/mbio.00360-26 (PMC13556257; doi:10.1128/mbio.00360-26)
Supplement: Supplementary file 1 [file mbio.00360-26-s0001.pdf]

The FleQ-FleN circuit balances flagellar number against fitness in *Pseudomonas aeruginosa*.

Anali Migueles-Lozano[1], Merrill Asp[4,5], Sofia T. Rocha[6,7], Jiaqi Li[8], Georgia Fanouraki[1], Aden D. Sun[1], Lichun Zhang[9], Jacob R. Waldbauer[9], Jiarong Hong[8], Abhishek Shrivastava[6,7], Jing Yan[4,5], Sampriti Mukherjee[1,2,3]*

[1]Department of Molecular Genetics & Cell Biology, University of Chicago, Chicago, IL, USA.

[2]Department of Microbiology, University of Chicago, Chicago, IL, USA.

[3]Duchossois Family Institute, University of Chicago, Chicago, IL, USA.

[4]Department of Molecular, Cellular and Developmental Biology, Yale University, New Haven, CT, USA.

[5]Yale Quantitative Biology Institute, Yale University, New Haven, CT, USA.

[6]School of Life Sciences, Arizona State University, Tempe, AZ, USA.

[7]Biodesign Institute, Arizona State University, Tempe, AZ, USA.

[8]Department of Mechanical Engineering, University of Minnesota, Minneapolis, MN, USA.

[9]Department of the Geophysical Sciences, University of Chicago, Chicago, IL, USA.

*Correspondence to: sampriti@uchicago.edu

SUPPLEMENTAL FIGURE LEGENDS

Supplemental Fig.1: **Monoflagellation is a robust trait in WT PA14 cells.** A) Percentages of cells with different flagella numbers through early exponential (OD_{600} = 0.25), exponential (OD_{600} = 0.5), early stationary (OD_{600} = 1) and late stationary (OD_{600} = 1.5) of WT (left) and Δ*fleN* (right). Grey: non-flagellated, Cyan: 1, Turquoise: 2, Dark turquoise: 3, Emerald green: 4, Dark green: More than 4 flagella per cell, 50 cells per replica, *n*=3. B) Percentage of cells with different flagellar placement from the cells in panel A), WT (left) and Δ*fleN* (right).

Supplemental Fig.2: Swimming area of *fleQ* mutants. A) Swimming assay of WT, Δ*fle*Q, Δ*fleN* and WT background cells carrying the FleQ point mutations from the Δ*fleN* suppressors. *n*=3. B) Measure area of swimming from A). Comparison shown only to WT. C) Growth curves of Δ*fleN attB::*P_{BAD}*fleN* strains under different arabinose concentrations. Error bars represent the SD of three biological replicates. D) Crystal Violet staining to measure biofilm formation in WT, Δ*fleN,* Δ*pelA* and $FleN^{V178G}$. Error bars represent the SEM of three biological replicates. For A) and D) statistical test used was One-way ANOVA, Turkey multiple comparisons test in GraphPad Prism software. Results were considered not statistically significant when p > 0.05.

Supplemental Fig.3: Representative micrographs for flagellar filament assembly and number (Alexa Flour 488, green) and membrane staining (FM4-64, red) of suppressor mutants of Δ*fleN*.

Supplemental Fig.4: Δ*fleN* suppressors class II flagellar genes expression. A) qPCR of fliF in wild-type, *ΔfleN, ΔfleQ;* and B) *ΔfleN* suppressors*.* Plotted values are the fold change of the ΔΔCq values. Statistical analysis and significance is based on the log2(fold change). Error bars represent SEM. Statistical analysis: One-way ANOVA, Dunnett's test in GraphPad Prism

software. **** P<0.0001, *** P <0.001, ** P <0.01. * P <0.05.

Supplemental Fig.5: **FleQ point mutations do not compromise stability of the protein.** A) Representative image of Western Blot of *fleQ* mutants (upper panel) and its quantification using ImageLab (lower panel), *n*=3. B) Alignment of *fleQ* AAA+-HTH domains amino acid sequence from different types of Pseudomonads (single and multiflagellated strains). In yellow, the *fleQ* mutations found in the suppressors of Δ*fleN* in PA14.

Supplemental Fig.6: **Δ*fleN* mutant cells viability and cell morphology.** A) Independent growth of WT and Δ*fleN* cells for 20hrs at 37°C in liquid, starting at the same OD_{600} (0.5). Colorimetric image of recovered colonies of each strain after 20hrs (left) and its respective quantification of CFU/ml (right), n=3. B) Length (left) and width (right) of WT and Δ*fleN* cells at OD_{600} = 0.5, 50 cells per replica, n=3. C) Nutrient competition between WT (green) and Δ*fleN* (red) cells. Representative image at the end of the competition (after 20hrs).

Supplemental Fig.7: Synteny of flagellar genes between *P. aeruginosa* PA14, *P. putida* KT2440 and *P. syringae pv. tomato* DC300. Hierarchical flagellar classes are denotated by the color of the arrows.

Supplemental Fig.8: Alignment of FleQ amino acid sequence from different types of Pseudomonads (single and multiflagellated strains).

Supplemental Fig.9: Alignment of FleN amino acid sequence from different types of Pseudomonads (single and multiflagellated strains).

Supplemental Fig.10: Alignment of FliC amino acid sequence from different types of Pseudomonads (single and multiflagellated strains).

SUPPLEMENTAL VIDEO LEGENDS

SV1: Flagellar wrapping affects bipolar multiflagellated motility. A Δ*fleN* cell (flagella in green, cell body in red) swims from left to right using flagella on the left pole. Flagella on the right pole are apparently bundled, as evidenced by one flagellum on the right pole unbundling at the end of the video, changing the cell's trajectory. 2.2 second duration. Real-time playback at 20 frames per second. 10 µm scale bar.

SV2: Hyperflagellation can enhance swimming speed. A Δ*fleN* cell (flagella in green, cell body in red) swims from lower left to upper right, increasing in speed as its multiple flagella cohere into a helical coil. Final speed exceeds 50 µm/s, higher than typical swimming speeds for monoflagellated wild-type cells. 2.15 second duration. Real-time playback at 20 frames per second. 10 µm scale bar.

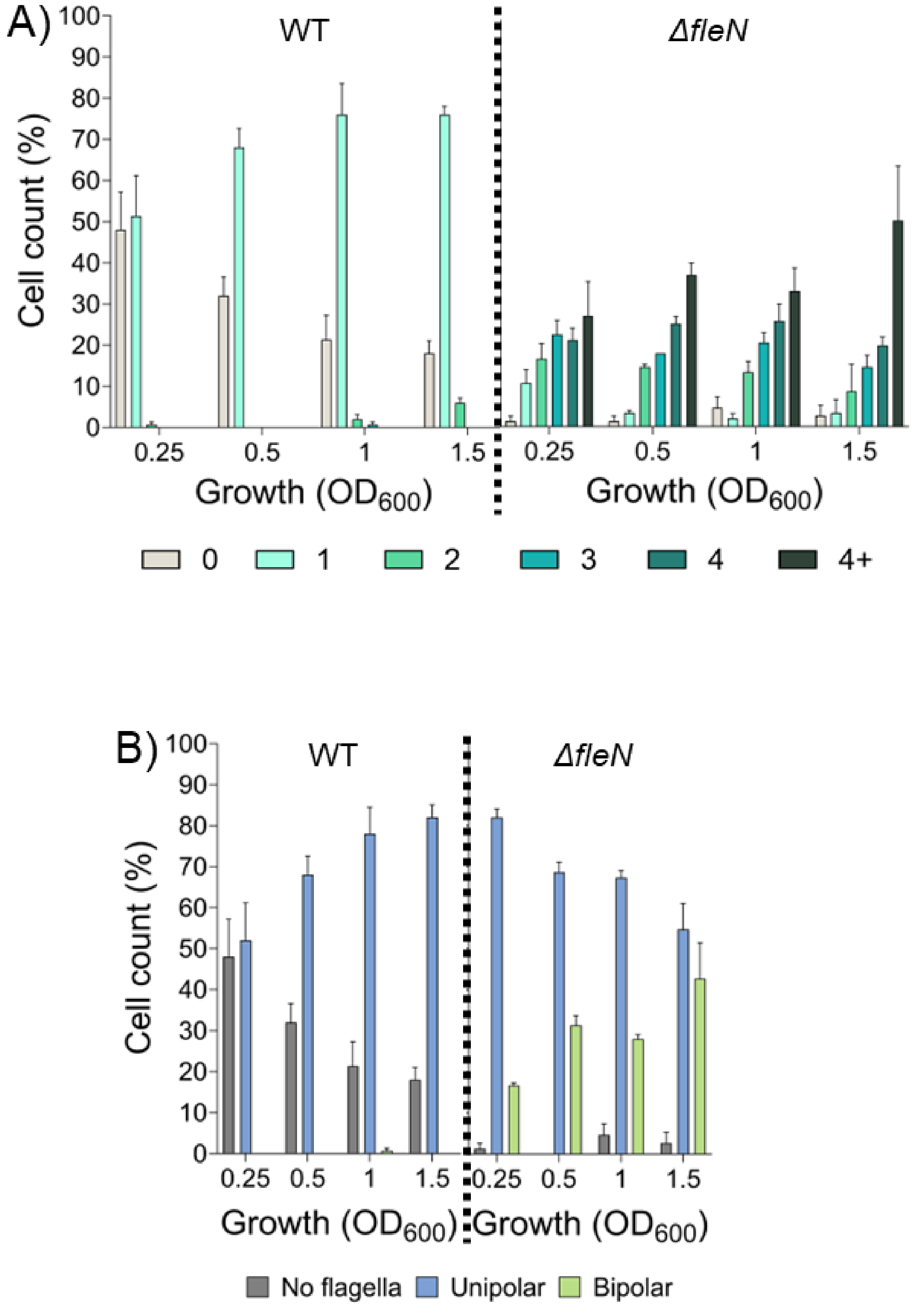
A)
WT
ΔfleN
Cell count (%)
Growth (OD600)
0
1
2
3
4
4+
B)
WT
ΔfleN
Cell count (%)
Growth (OD600)
No flagella
Unipolar
Bipolar

Supplemental Figure 2

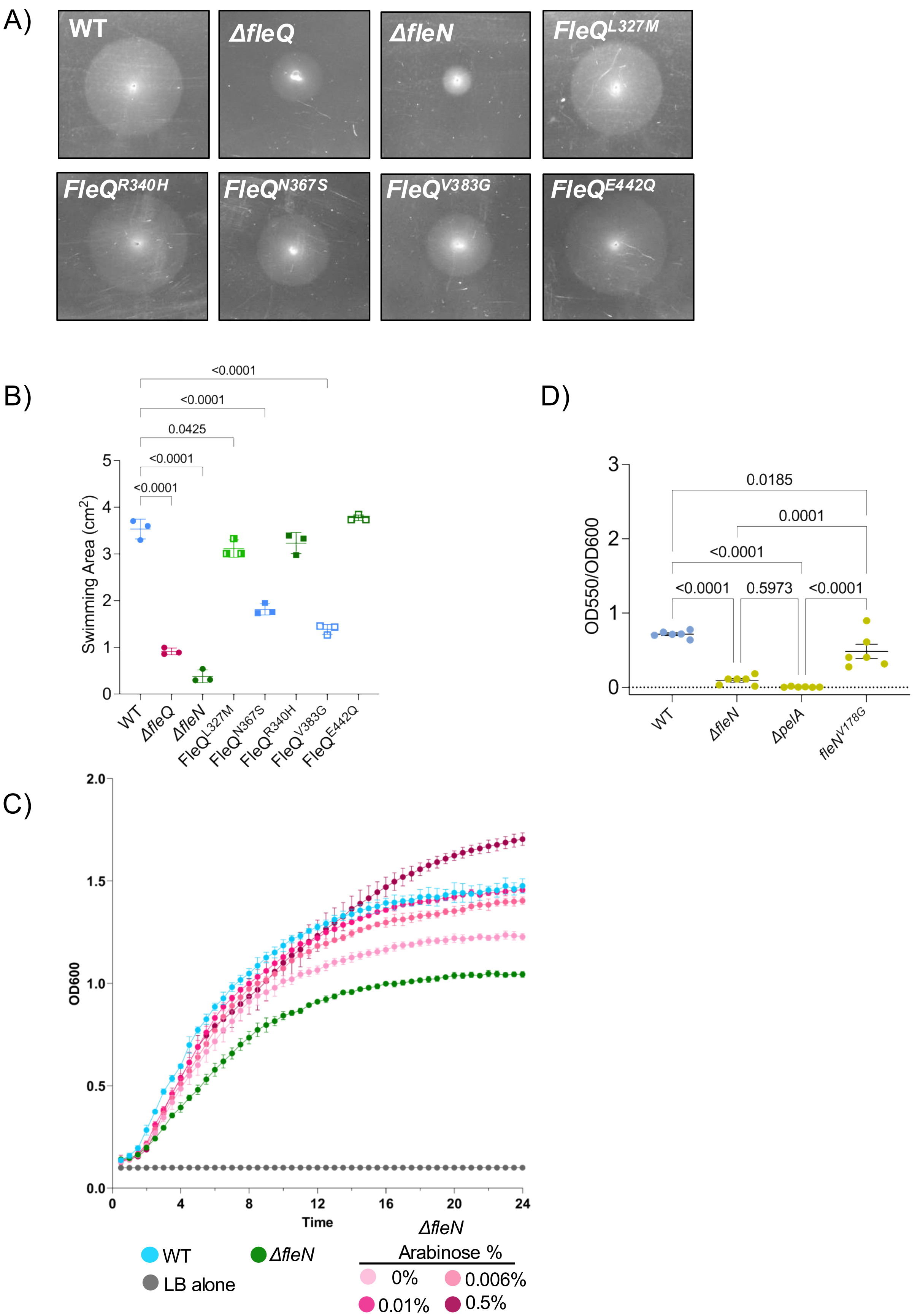

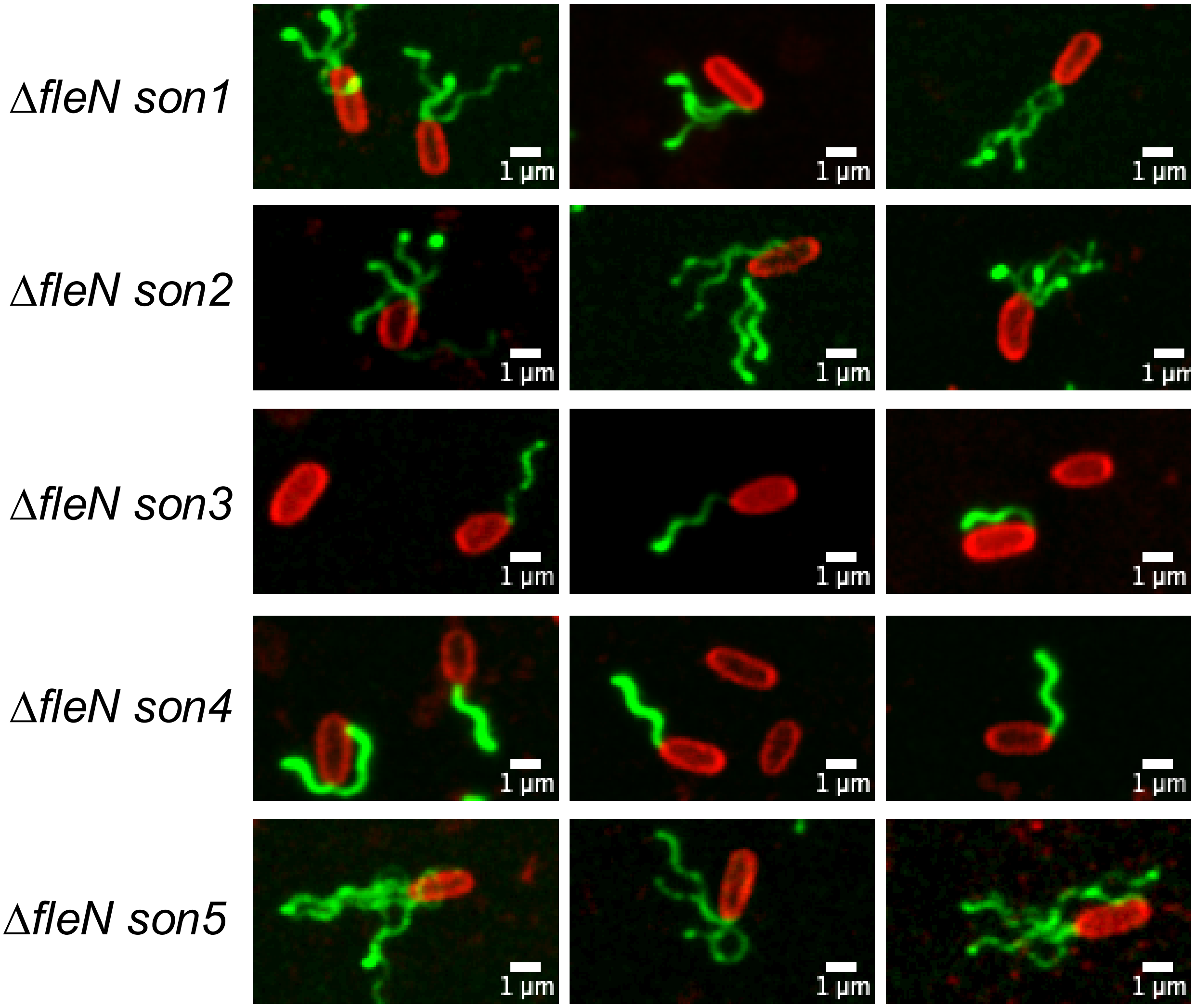
ΔfleN son1
ΔfleN son2
ΔfleN son3
ΔfleN son4
ΔfleN son5
1 μm

A)

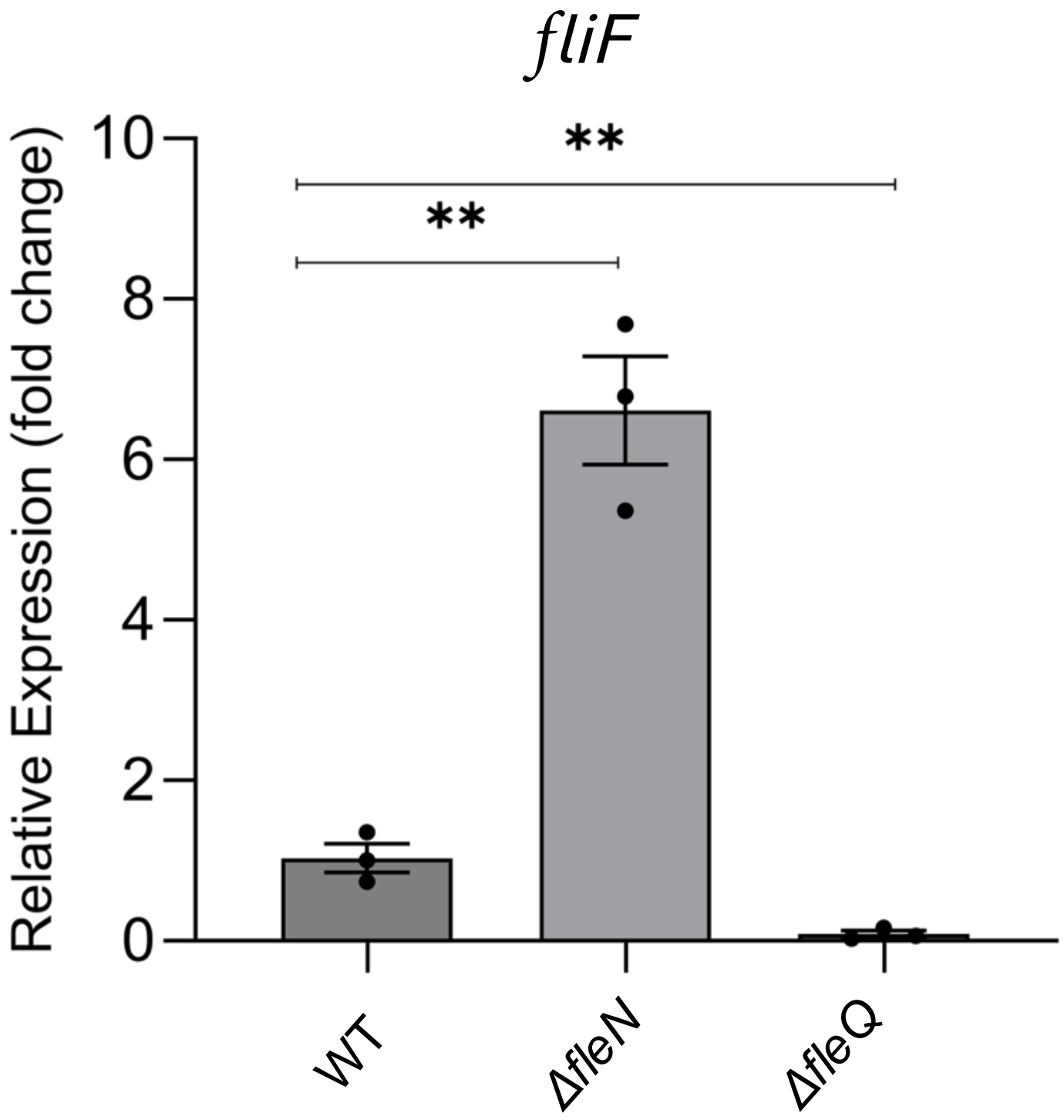
fliF
**
**
Relative Expression (fold change)
10
8
6
4
2
0
WT
ΔfleN
ΔfleQ

B)

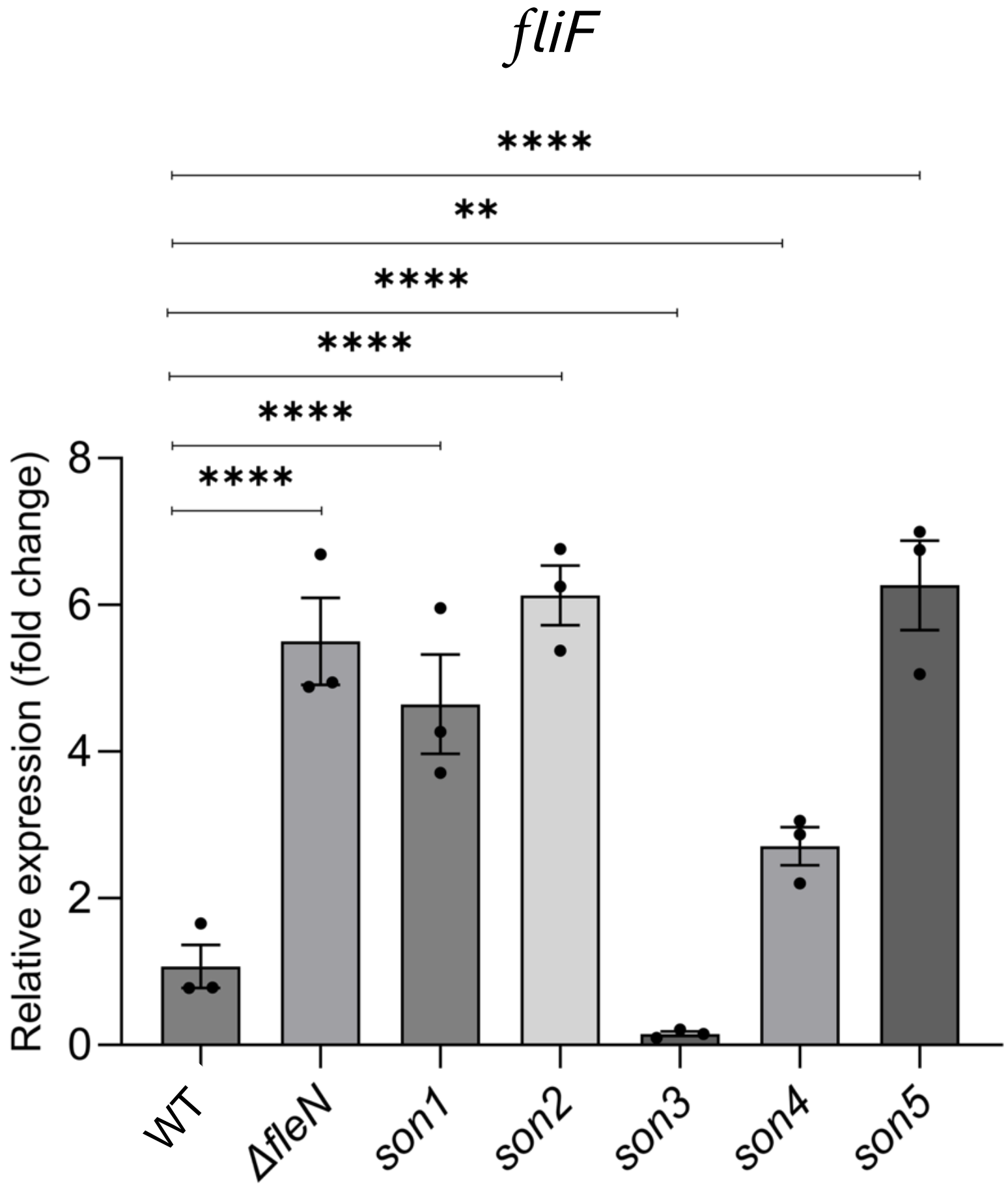
fliF
****
**
****
****
****
****
Relative expression (fold change)
8
6
4
2
0
WT
ΔfleN
son1
son2
son3
son4
son5

Supplemental Figure 5

A)

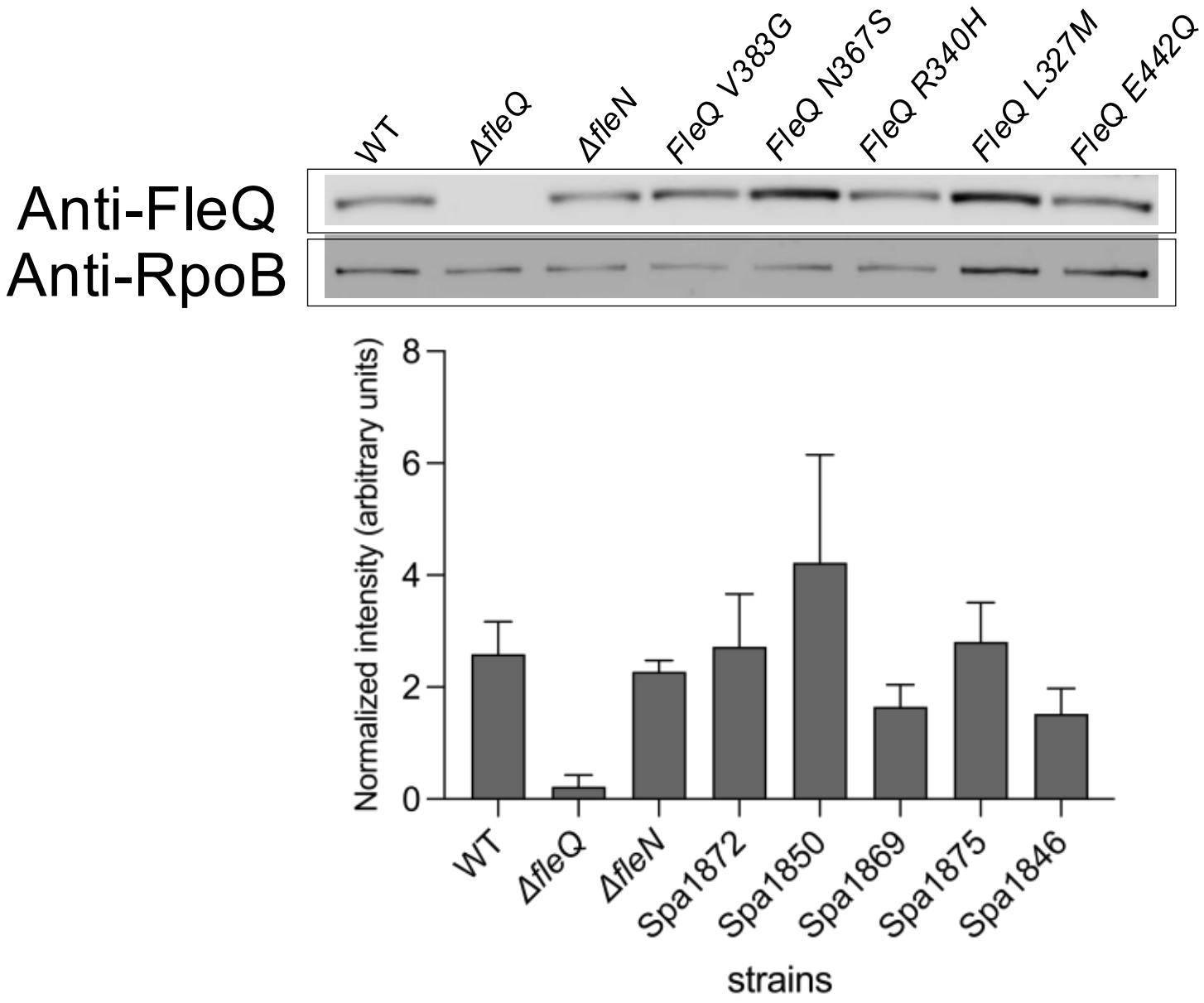

B)

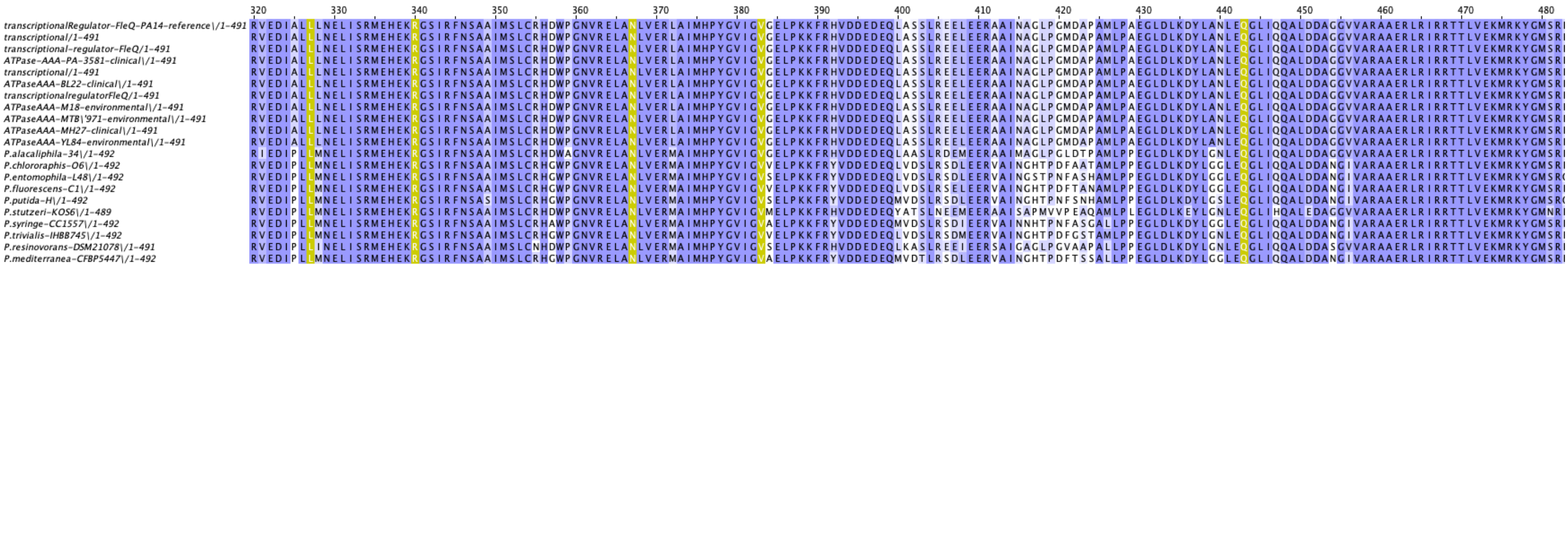

Supplemental Figure 6

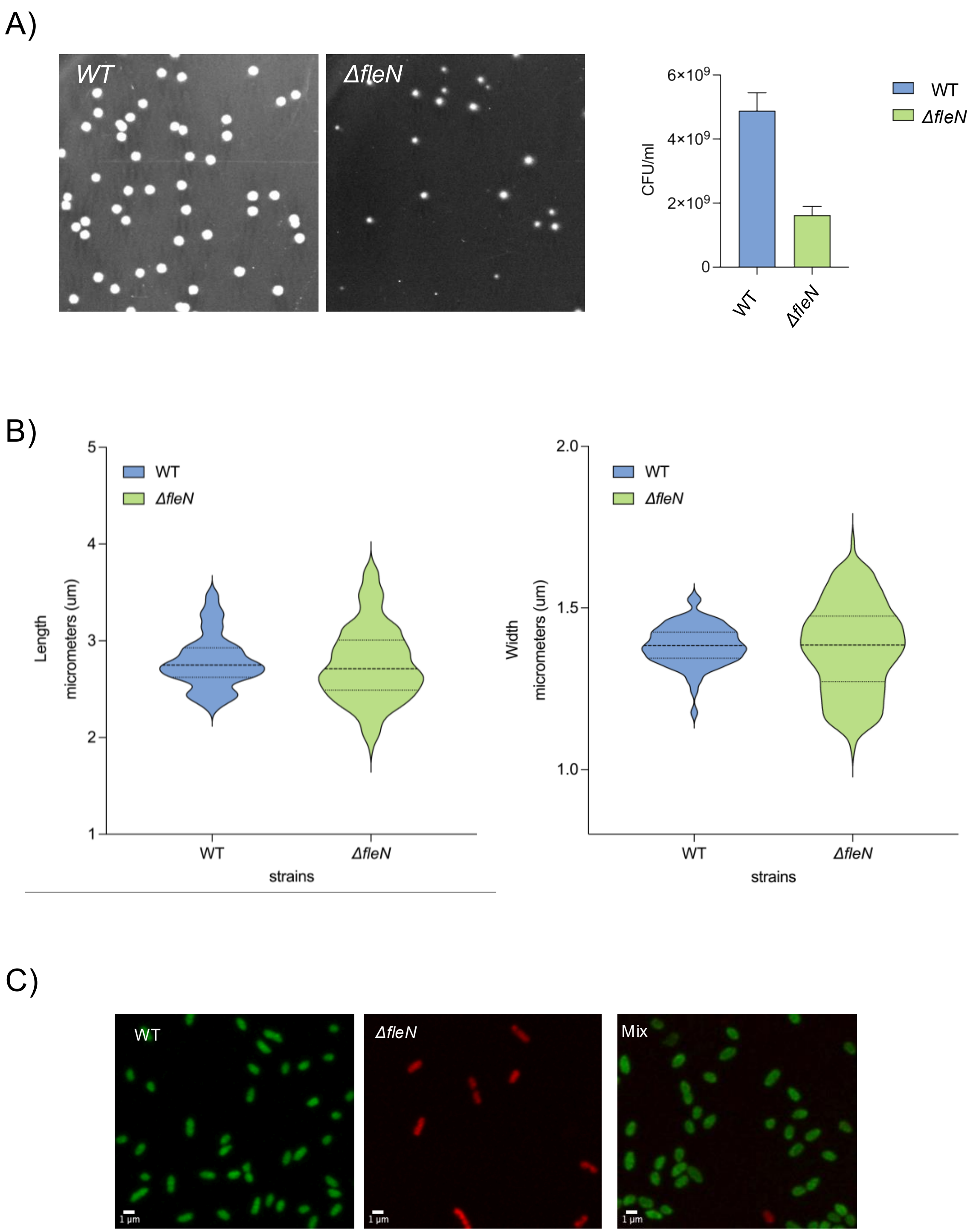

Supplemental Figure 7

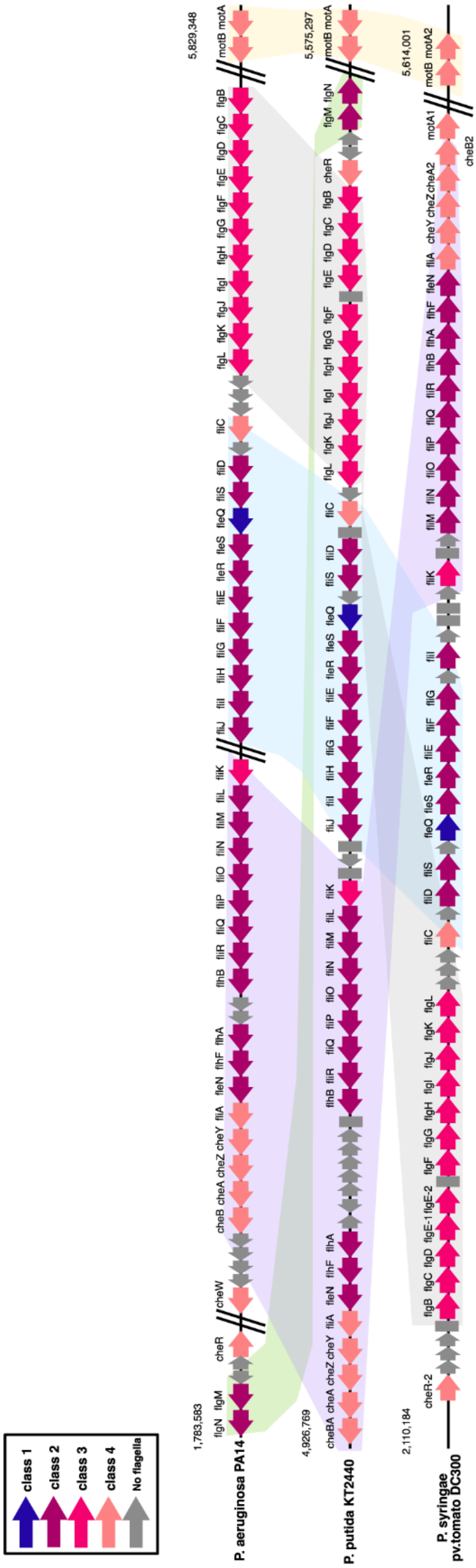

FleQ

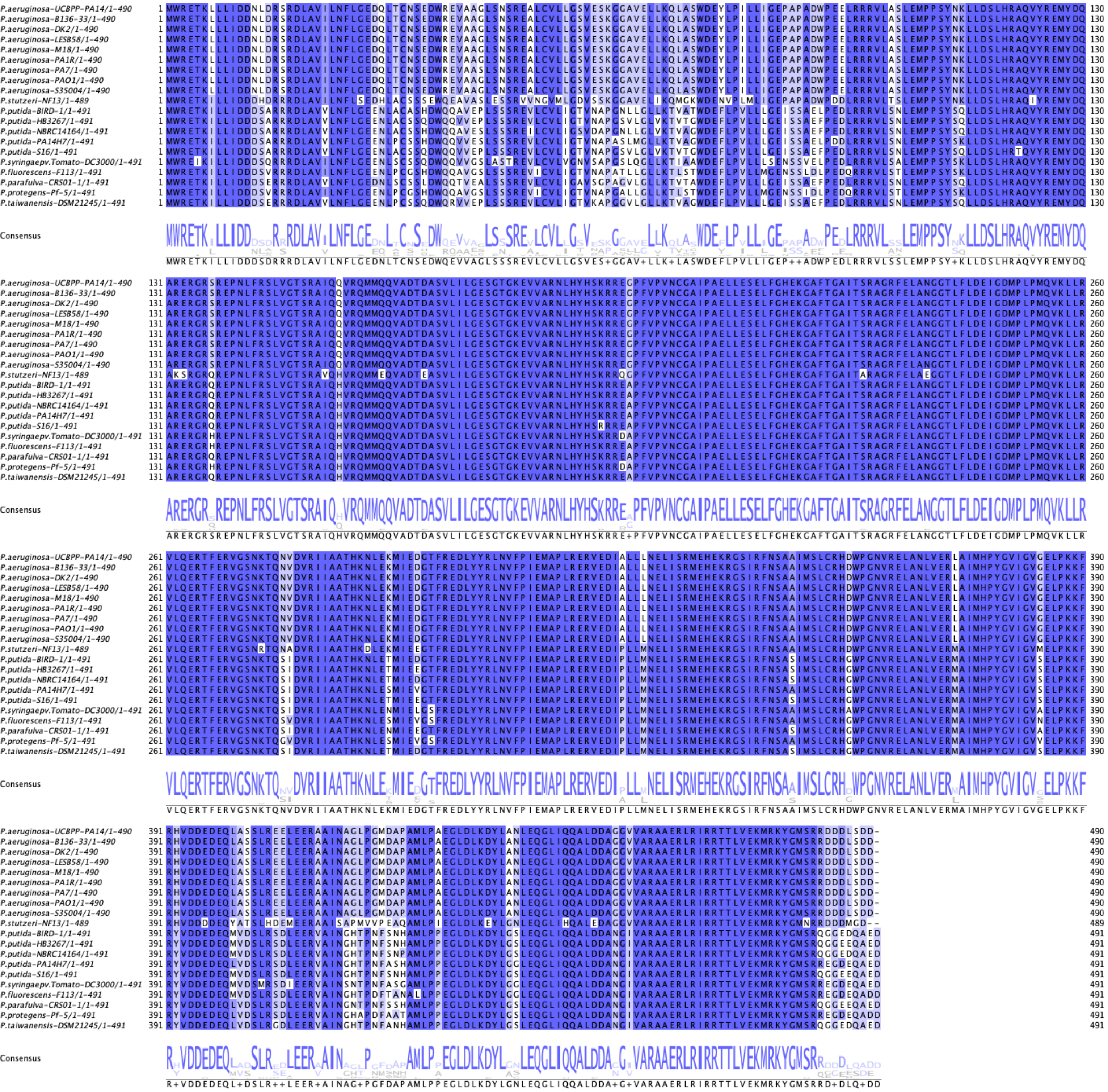
P.aeruginosa-UCBPP-PA14/1-490
P.aeruginosa-B136-33/1-490
P.aeruginosa-DK2/1-490
P.aeruginosa-LESB58/1-490
P.aeruginosa-M18/1-490
P.aeruginosa-PA1R/1-490
P.aeruginosa-PA7/1-490
P.aeruginosa-PAO1/1-490
P.aeruginosa-S35004/1-490
P.stutzeri-NF13/1-489
P.putida-BIRD-1/1-491
P.putida-HB3267/1-491
P.putida-NBRC14164/1-491
P.putida-PA14H7/1-491
P.putida-S16/1-491
P.syringaepv.Tomato-DC3000/1-491
P.fluorescens-F113/1-491
P.parafulva-CRS01-1/1-491
P.protegens-Pf-5/1-491
P.taiwanensis-DSM21245/1-491
Consensus

FleN

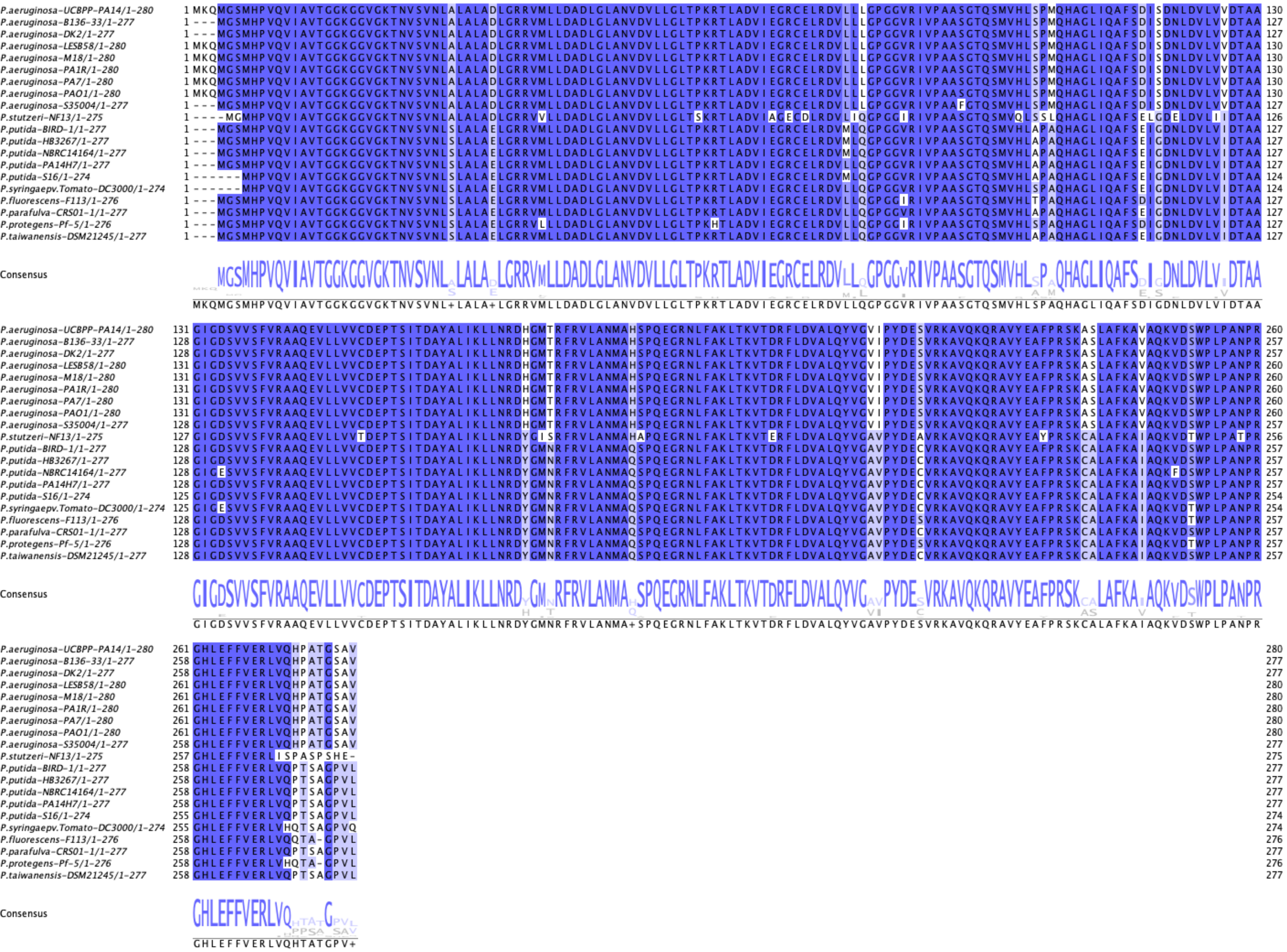
P.aeruginosa-UCBPP-PA14/1-280
P.aeruginosa-B136-33/1-277
P.aeruginosa-DK2/1-277
P.aeruginosa-LESB58/1-280
P.aeruginosa-M18/1-280
P.aeruginosa-PA1R/1-280
P.aeruginosa-PA7/1-280
P.aeruginosa-PAO1/1-280
P.aeruginosa-S35004/1-277
P.stutzeri-NF13/1-275
P.putida-BIRD-1/1-277
P.putida-HB3267/1-277
P.putida-NBRC14164/1-277
P.putida-PA14H7/1-277
P.putida-S16/1-274
P.syringaepv.Tomato-DC3000/1-274
P.fluorescens-F113/1-276
P.parafulva-CRS01-1/1-277
P.protegens-Pf-5/1-276
P.taiwanensis-DSM21245/1-277
Consensus

Supplemental Figure 10

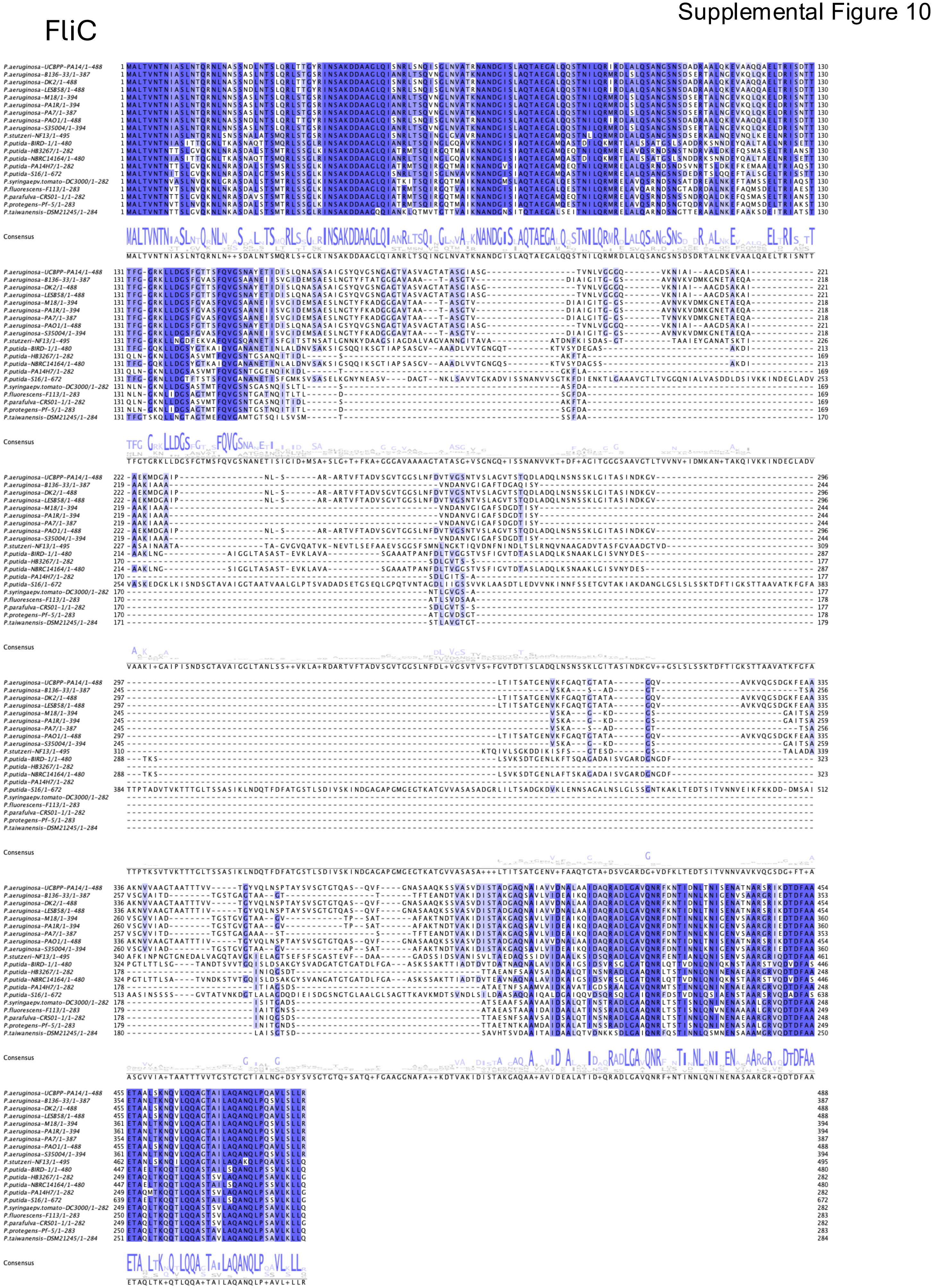

SUPPLEMENTAL TABLES

TABLE S1: Unique suppressor mutations of the Δ*fleN* motility phenotype and WT hyperswimmers.

PA14 ID[a]	Gene name	Nucleotide position	Mutation	Occurrence
PA14_50220	*fleQ*	4461021	*E442Q (GAG->CAG)*	2/10
PA14_50220	*fleQ*	4461245	*N367S (AAC->AGC)*	2/10
PA14_50220	*fleQ*	4461326	*R340H (CGC->CAC)*	3/10
PA14_50220	*fleQ*	4461197	*V383G (GTC->GGC)*	2/10
PA14_50220	*fleQ*	4461366	*L327M (CTG->ATG)*	1/10
PA14_45640	*fleN*	4059699	*V178G (GTC->GGC)*	8/8

a: annotation from www.pseudomonas.com (Winsor et al., 2016)

TABLE S2: Bacterial strains and plasmids

Strain	Description	Reference
UCBPP-PA14	Wild type *Pseudomonas aeruginosa*	Laboratory stock
SPa0739	*fliC*^{T394C}	This study
SPa1571	*fliC*^{T394C}, *ΔfleN*	This study
SPa1825	*ΔfleQ, fliC*^{T394C}	This study
SPa1821	*ΔfliC*	This study
SPa1857	*ΔfleN, attB::P*$_{araBAD}$*-fleN*	This study
SPa1839	*fleQ*^{E442Q}, *fliC*^{T394C}	This study
SPa1902	*fleQ*^{N367S}, *fliC*^{T394C}	This study
SPa1901	*fleQ*^{R340H}, *fliC*^{T394C}	This study
SPa1854	*fleQ*^{V383G}, *fliC*^{T394C}	This study
SPa1842	*fleQ*^{L327M}, *fliC*^{T394C}	This study
SPa1846	*fleQ*^{E442Q}, *ΔfleN, fliC*^{T394C}	This study
SPa1850	*fleQ*^{N367S}, *ΔfleN, fliC*^{T394C}	This study
SPa1869	*fleQ*^{R340H}, *ΔfleN, fliC*^{T394C}	This study
SPa1872	*fleQ*^{V383G}, *ΔfleN, fliC*^{T394C}	This study
SPa1875	*fleQ*^{L327M}, *ΔfleN, fliC*^{T394C}	This study
SPa1926	*ΔfleN, fliC*^{T394C}, *PA14_20530-3'IGR::P*$_{hyspank}$*-mkate2*	This study
SPa0755	*fliC*^{T394C}, *PA14_20530-3'IGR::P*$_{hyspank}$*-mkate2*	This study
SPa0157	*fliC*^{T394C}, *PA14_20530-3'IGR::P*$_{hyspank}$*-mNeonGreen*	This study
SPa1922	*fleQ*^{E442Q}, *ΔfleN, fliC*^{T394C}, *PA14_20530-3'IGR::P*$_{hyspank}$*-mkate2*	This study
SPa1923	*fleQ*^{R340H}, *ΔfleN, fliC*^{T394C}, *PA14_20530-3'IGR::P*$_{hyspank}$*-mkate2*	This study
SPa1852	*ΔfleN, ΔfliC*	This study
SPa1947	*ΔfliF*	This study
SPa1949	*ΔfleN, ΔfliF*	This study
SPa0239	*ΔpelA*	This study

SPa2034	*fleN*^{V178G} isolate	This study
SEc0646	SM10 λpir pEXG2-Δ*fleN*	This study
SEc0081	SM10 λpir pEXG2-Δ*fliC*	This study
SEc1041	SM10 λpir pEXG2_Δ*fliF*	This study
SEc0932	SM10 λpir pEXG2-Δ*fleQ*	This study
SEc0099	SM10 λpir pEXG2-*fliCT394C*	This study
SEc0975	SM10 λpir pEXG2-*fleQ*^{E442Q} from isolate	This study
SEc0980	SM10 λpir pEXG2- *fleQ*^{N367S} from isolate	This study
SEc0976	SM10 λpir pEXG2- *fleQ*^{R340H} from isolate	This study
SEc0977	SM10 λpir pEXG2- *fleQ*^{V383G} from isolate	This study
SEc0979	SM10 λpir pEXG2- *fleQ*^{L327M} from isolate	This study
SEc0955	DH5α pTJ1-*fleN*	This study
SEc0059	HB101 pRK2013	Laboratory stock
SEc0057	DH5α pTNS3	Laboratory stock
E.coli HB101		Laboratory stock
E.coli DH5α	F^{-} *endA1 glnV44 thi-1 recA1 relA1 gyrA96 deoR nupG purB20* φ80d*lacZ*ΔM15 Δ(*lacZYA-argF*) U169, hsdR17($r_K^-m_K^+$), λ^{-}	Laboratory stock
E.coli SM10λ*pir*	*thi thr leu tonA lacY supE recA*::RP4-2-Tc::Mu	Laboratory stock
pEXG2	Allelic exchange vector with pBR origin, gentamicin resistance, *sacB* gene	(Hmelo et al., 2015)
pTJ1	*E. coli-Pseudomonas* Amp^{r} shuttle vector	Laboratory stock
pRK2013	*E. coli-Pseudomonas* Kan^{r} shuttle vector	Laboratory stock
pTNS3	*E. coli-Pseudomonas* Amp^{r} shuttle vector	Laboratory stock

TABLE S3: Primer List

Primer No.	Name	Sequence
SP2343	DfleQ UP F-pEXG2	AGGTCGACTCTAGAGGATCCATGGAGCGGGAGCAGATCC
SP2344	DfleQ UP R	GGTTTCGCGCCACATTTTGATCAG
SP2345	DfleQ DN F	GATCAAAATGTGGCGCGAAACCGACCTGTCGGATGATTGACAGGTC
SP2346	DfleQ DN R-pEXG2	TCATGCGCACCCGTGGAAAGGCGAACACCAGCATGTCG
SP1214	pEXG2-ITA-1	GGATCCTCTAGAGTCGACCTGC
SP1215	pEXG2-ITA-2	TTTCCACGGGTGCGCATGATC

AML_p_280	FleQ-Sanger3-F	CCAGCCCTCTTTCGGGTCGA
AML_p_281	FleQ-Sanger4-F	CCGCTGCCGATGCAGGTCAA
SP2175	DfleN UP F-pEXG2	GGTCGACTCTAGAGGATCCGTACCGGTGACCCTGGTCGATC
SP2176	DfleN UP R	ACCCATCTGCTTCATACCTTGTGTTG
SP2177	DfleN DN F	AAGGTATGAAGCAGATGGGTTTCGTCGAACGACTGGTCCAAC
SP2178	DfleN DN R-pEXG2	ATCATGCGCACCCGTGGAAAGCTCCGGCAACTTGGCGATG
SP2378	pTJ1-fleN F ITA	AGGAGATATACATACCCATGATGAAGCAGATGGGTAGCATGC
SP2379	pTJ1-fleN R ITA	CGGGTACCGAGCTCGAATTCTCATACGGCCGAACCTGTCG
SP1822	pTJ1 F ITA	GAATTCGAGCTCGGTACCCG
SP1823	pTJ1 R ITA	CATGGGTATGTATATCTCCTTCTTAAAG
DM155	glmS_UP2_F	GGTACTCGCCTCGCGCTCC
DM156	glmS_DW2_R	GTGGCGCCGAACAACGAGC
AML_p_293	Down-FliF-F-pEXG2	CGGCGCGAGGATAACTAGTTCCTCCTGCCGAGCCCCACGG
AML_p_294	Down-FliF-R	GATCATGCGCACCCGTGGAAACAGCAGCTCGGCGGCACGC
AML_p_295	UP-FliF-R	CTCCGTGGGGCTCGGCAGGAGGAACTAGTTATCCTCGCGCCGCTCAG
AML_p_296	UP-FliF-F-pEXG2	GCAGGTCGACTCTAGAGGATCCCATGCCTGGCCGGGCAACG
SP399	Physpank R	TATGGATCCCTCCTTCCAGCTAG
SP262	ectsite3 DN F	CTGATGAGGCTGGTATTTCAATTG
SP1216	pEXG2-ect3 F	GCTTCTGCAGGTCGACTCTAGAGGATCCAGCGGCTATCTTTACTCAAAGAG

SP1217	pEXG2-ect3 R	ACGATCATGCGCACCCGTGGAAATGCTCGGGAAAGAAAGGCGGTATC
SP376	mkate2-ect3 R	CAATTGAAATACCAGCCTCATCAGTTATCATCTGTGCCCCAGTTTGCTAGG
SP402	Physpank-mkate2 F	GTCGACAGCTAGCTGGAAGGAGGGATCCATAATGGTGAGCGAGCTGATTAAGG
SP2158	mNG-ect3DN ITA F	TGGACGAGCTGTACAAGTAATGACTGATGAGGCTGGTATTTCAATTG
